# Mendel and Darwin: untangling a persistent enigma

**DOI:** 10.1038/s41437-019-0289-9

**Published:** 2019-12-17

**Authors:** Daniel J. Fairbanks

**Affiliations:** Department of Biology, Utah Valley University, 800 W. University Parkway, Orem, UT 84058 USA

**Keywords:** Evolutionary biology, Evolutionary theory

## Abstract

Mendel and Darwin were contemporaries, with much overlap in their scientifically productive years. Available evidence shows that Mendel knew much about Darwin, whereas Darwin knew nothing of Mendel. Because of the fragmentary nature of this evidence, published inferences regarding Mendel’s views on Darwinian evolution are contradictory and enigmatic, with claims ranging from enthusiastic acceptance to outright rejection. The objective of this review is to examine evidence from Mendel’s published and private writings on evolution and Darwin, and the influence of the scientific environment in which he was immersed. Much of this evidence lies in Mendel’s handwritten annotations in his copies of Darwin’s books, which this review scrutinises in detail. Darwin’s writings directly influenced Mendel’s classic 1866 paper, and his letters to Nägeli. He commended and criticised Darwin on specific issues pertinent to his research, including the provisional hypothesis of pangenesis, the role of pollen in fertilisation, and the influence of “conditions of life” on heritable variation. In his final letter to Nägeli, Mendel proposed a Darwinian scenario for natural selection using the same German term for “struggle for existence” as in his copies of Darwin’s books. His published and private scientific writings are entirely objective, devoid of polemics or religious allusions, and address evolutionary questions in a manner consistent with that of his scientific contemporaries. The image that emerges of Mendel is of a meticulous scientist who accepted the tenets of Darwinian evolution, while privately pinpointing aspects of Darwin’s views of inheritance that were not supported by Mendel’s own experiments.

## Introduction

Gregor Mendel and Charles Darwin are arguably the two most influential nineteenth-century founders of modern biology. Darwin’s theory of evolution through natural selection revolutionised the science of his day and beyond, trumpeted by his supporters and vilified by his detractors from the time it was published to the present. Mendel’s theory, by contrast, lay mostly dormant until the dawn of the twentieth century when it rapidly drew both fame and criticism through its dramatic rediscovery. Mendel and Darwin were contemporaries, yet the path connecting them during their lifetimes was entirely a one-way street: Mendel was familiar with Darwin’s books, having read and annotated German translations of them, whereas all available evidence indicates that Darwin knew nothing of Mendel.

Much of Darwin’s writings, both published and private, are remarkably well-conserved and documented, providing a plethora of evidence of his activities and thoughts throughout his life. The near opposite is the case for Mendel. Documentation is sparse and fragmentary, much of it lost or destroyed, some of it circumstantial and second-hand. This scarcity of evidence has led to considerable controversy (as reviewed by Fairbanks and Rytting 2001). Most notable is the Mendel–Fisher controversy, a decades-long dispute over the statistical integrity of Mendel’s data, which is now essentially resolved (Franklin et al., 2008). In contrast, the Mendel–Darwin connection, and Mendel’s views on evolution in general and Darwin in particular, remain enigmatic, often shrouded in myth and conjecture. In this review, I will attempt to untangle the evidence, both direct and circumstantial, regarding Mendel’s approach to evolution generally, and to Darwin specifically, including evidence that has recently come to light.

## Mendel’s pre-Darwinian views on evolution (1822–56)

Johann Mendel was born in 1822 in Hynčice (Heizendorf)^1^ in Austrian Silesia, currently in Czechia (the Czech Republic). At great sacrifice to his family, he attended boarding schools until the age of 21. He was academically successful, and in 1843 was admitted to the Augustinian Order of the St. Thomas Monastery in Brno (Brünn), where he adopted the name Gregor.

From that time forward, he found himself in extraordinary intellectual surroundings. He was a friar, not a monk, in that the Augustinian friars of his monastery served the public by openly interacting with the local people, pursuing advanced education in secular subjects, participating in scholarly societies, serving in civic roles, pursuing research and writing, and teaching their academic specialties in schools. The consequence of Mendel’s attempt to serve as a priest is best described in the words of his abbot, Cyrill Napp: “he is very diligent in the study of the sciences; but…is much less fitted for work as a parish priest, the reason being that he is seized by an unconquerable timidity…[which] is why I found it necessary to relieve him from service as a parish priest” (Iltis, 1966, p. 58). Mendel’s timidity would resurface numerous times, influencing his scientific pursuits in both positive and negative ways.

### Mendel as a science teacher

A fortuitous consequence of Mendel’s timidity was the fact that it motivated Napp to assign him to teach natural sciences in Znojmo (Znaim), southwest of Brno. This assignment freed him to pursue science with students, unencumbered by parish-priest duties. He was a superb and popular teacher, though not certified. In November 1849, the Austrian government issued an edict that all teachers must be certified through a university examination (Klein and Klein 2013). The University of Vienna would administer Mendel’s examination, consisting of an off-site written portion (referred to as homework), and on-site written and oral sessions.

In the summer of 1850, Mendel received the homework portion wherein we encounter his first words alluding to evolution:

In the course of time, when the earth had attained the capability necessary for the formation and preservation of organic life, plants and animals of the lowest species first appeared. The period of organic formation was not infrequently interrupted by catastrophes, which threatened the life of organisms and, in part, led to their decline. … The vegetable and animal life developed more and more richly; its oldest forms disappeared in part to make way for new and more perfect ones.

(Orel et al. 1983, p. 237*^2^).

The influence of old-earth catastrophism on Mendel’s thought is evident, probably from Karl Hermann Konrad Burmeister’s (1843) book *Die Geschichte der Schöpfung* (*The History of Creation*), which Mendel cited. Given Mendel’s acceptance of old-earth catastrophism at this early stage of his career, it is unclear whether he viewed descent from surviving species, as opposed to de novo creation, as the mechanism for the emergence of new species. Notably, Orel et al. (1983) and Klein and Klein (2013) pointed out that Mendel used the words “zum Theile”, translated as “in part”, referring to life declining, leaving open the interpretation that new species arose from those that survived catastrophes. In any case, he was about to enter one of Europe’s most active and contentious pre-Darwinian evolutionary environments, where evolution by descent over eons of geologic time was the underlying theme. There is no evidence that he ever returned to catastrophism.

Mendel’s examiners in physics, Andreas von Baumgartner and Christian Doppler, praised his physics essay. The examiner for geology and natural history, Rudolph Kner, determined that Mendel’s account and sources were inadequate and outdated. Nonetheless, Mendel was invited for the written and oral sessions on-site at the University of Vienna. He exceled in some portions and failed others, and thus did not pass the certification examination. Nonetheless, his examiners agreed that he was well-prepared for university studies, allowing him to attend the University of Vienna. According to Orel (1996): “Had he passed his teachers’ examination, he would have stayed on at the Znojmo *Gymnasium*, and several generations of secondary school pupils would have gained an excellent teacher. Science, on the other hand, would almost certainly have lost one of its leading discoverers” (p. 66).

### At the University of Vienna

In November 1851, Mendel enroled at the University of Vienna as an “extraordinary” student, meaning that he did not pursue a degree, audited his classes, and was not formally evaluated (Klein and Klein 2013). Over a period of 22 months (November 1851 to August 1853), he enroled in physics, chemistry, mathematics, zoology, botany, and palaeontology courses, several with evolutionary content. His studies could hardly have been timed better to coincide with a surge of evolutionary fervour that would dramatically engage Vienna’s intellectual and religious communities. One of Mendel’s botany professors, Franz Unger, was the key player in this surge. By 1851 Unger vigorously promoted what Gliboff (1999) called “Unger’s theory of universal common descent” (p. 223), viewing all new species as arising from previous species, and, in Unger’s words, “not by any means a one-sided lineal progression, but a radiation broadening out on all sides” (Unger 1853, p. 107).

From May through October of 1851, Unger popularised evolution through a series of seventeen articles under the title *Botanische Briefe* (*Botanical Letters*) in the *Wiener Zeitung* (*Vienna News*), later compiled into a book (Unger 1852). Unger popularised how the fossil record had revealed plant and animal evolution over eons of geological time. The 17th and final instalment was published on October 18, 1851, just nine days before Mendel arrived in Vienna (Gliboff 1998).

Unger espoused the view that new species emerged from previous ones in a passage that is remarkable in foretelling the theme of genetic recombination in evolution that Mendel would address in his research (as noted by Olby 1985; Orel 1996; Klein and Klein 2013): “who can deny that new combinations of the elements arise out of this permutation of vegetation ever reducible to a certain law—combinations which emancipate themselves from the preceding characteristics of the species, and appear as new species?” (Unger 1853, p. 92). That same year, Unger (1851) also published a momentous popular book on evolution, *Die Urwelt in ihren verschiedenen Bildungsperioden* (*The Primitive World in its Various Transitional Periods*), which was well received in Vienna (Gliboff 1998).

Unger’s publications drew the ire of a prominent Roman Catholic priest, Sebastien Brunner, founding editor of the *Wiener Kirchenzeitung* (*Vienna Church News*), who published several editorials ridiculing him. The first appeared on October 25, 1851, just two days before Mendel’s arrival. Brunner claimed (in excerpts translated by Olby 1985) that, “paganism is taught in the universities in all branches of science” (p. 201), that Unger was “a man who openly denied the creation and the Creator” (p. 202), and that “professors at so-called Catholic universities deliver lectures on really beastly theories for years on end” (p. 203). Mendel undoubtedly was aware of this very public dispute, yet enroled in Unger’s classes in 1852 possibly placing him in a precarious position as a member of the clergy.

### Return to Brno and ecclesiastical condemnation

In August 1853, Mendel returned to Brno, still uncertified as a science teacher but well trained in scientific theory and methodology. He immediately encountered an environment of official censure for secular pursuits, not unlike the ecclesiastical condemnation of science he had witnessed in Vienna. The Bishop of Brno, Anton Ernst Schaffgotsch, had been assigned to investigate the Augustinian monasteries in Prague, Krakow, and Brno for secularism, beginning in 1853 while Mendel was still in Vienna (Orel 1996; Klein and Klein 2013). In 1854, after Mendel had returned, Schaffgotsch conducted an on-site investigation. In his report, recommending that the monastery be disbanded, he wrote (as translated by Klein and Klein 2013): “In a word, in the house tending the Rule of St. Augustine reigns a secular spirit which the few lappets of the Augustinian habit fail to cover up”, and specifically of Mendel, “he studies profane sciences at a worldly institution in Vienna at the expense of the monastery to become a professor of said sciences at a state institution” (p. 295).

In the meantime, Brunner’s attacks reached their finale in early 1856 in an editorial titled “Isis Priest and Philistine”, specifically directed at Unger and his colleagues: “the modern high priests of Isis, that is to say holders of chairs in various branches of natural history, seldom or never enter a church—according to their pronouncements, nature is their temple”*. Next, Brunner satirises Vienna’s botanists: “they do everything they can to make themselves into plants of botanical learning that can be smelt from afar—and place themselves voluntarily into the eternally stinking dung-bed of the pantheistic world-view, which nevertheless fosters a certain richness of blossoms”*.

In both Vienna and Brno, the environment pitting the church against evolution had become exceedingly vitriolic. As a cleric enduring overt reproach from the Bishop of Brno, and who had studied under an evolutionist as infamous as Unger, Mendel undoubtedly suffered from burdensome conflictions. Unfortunately, none of his surviving writings reveal anything about how he personally viewed this dilemma.

Just weeks after Unger’s dispute with Brunner had reached its pinnacle, Mendel travelled to the University of Vienna to sit for a second teacher-certification examination, on May 5, 1856. One of his fellow friars recounted what transpired: “Although he fortunately received easy questions, he fell ill after the first one, so that it was impossible for him to write. He seems, in any case, to be suffering from nerves. … Fearing further similar attacks, he returned home without having accomplished anything” (Kříženecký 1963, p. 308*). To what extent the conflicting loyalties Mendel felt to the church and to his scientific integrity contributed to this nervous breakdown can only be surmised. The immediate result was devastating. Mendel was confined to his bed, and word sent to his family prompted his ailing father and brother-in-law to travel to Brno to attend to him (Iltis 1966, Orel 1996, Klein and Klein 2013). The long-term result was also demoralising. In spite of his ambitions, he would never attain certification as a science teacher.

### Mendel’s classic paper and Darwin’s initial influence (1856–66)

The role of hybridisation in the evolution of new species was one of the most extensively researched topics during Mendel’s lifetime. Two years before Mendel entered the University of Vienna, Carl Friedrich von Gärtner (1849) published his magnum opus, *Versuche und Beobachtungen über die Bastarderzeugung im Pflanzenreiche* (*Experiments and Observations on Hybrid Production in the Plant Kingdom*), which Mendel obtained, probably while at university. His personal copy contains his annotations (Olby 1985), and he referred to it often in his classic paper. Darwin (1859) likewise considered the topic important, devoting an entire chapter to hybridism in *Origin of Species*.

Mendel began his hybridisation experiments with garden pea (*Pisum sativum*) at about the same time as his second teacher-certification examination, in the spring of 1856. He had planned well for these experiments, spending the two previous summers (1854 and 1855) growing and examining the varieties he would choose as parents to ensure that they were true-breeding. His classic paper, “Versuche über Pflanzen Hybriden” (“Experiments on Plant Hybrids”) would be published a decade later (Mendel 1866).

At the encouragement of Charles Lyell, Darwin began writing what he intended to be a massive multi-volume work on natural selection this same spring, on May 14, 1856 (Darwin 1856, Darwin 1887, p. 67). Two years later he would begin reorganising and rewriting material from this manuscript to produce several books, including the two that most influenced Mendel: *Origin of Species* (1859) and *The Variation of Animals and Plants Under Domestication* (1868).

*Origin of Species* first appeared in November 1859 (Darwin 1859), and the first German translation in 1860 (Darwin 1860). Mendel owned a second-edition German translation published in 1863 (Darwin 1863; Orel 1971b; Fairbanks and Rytting 2001). Since Mendel completed his pea hybridisation experiments this same year, he probably read and annotated his copy of *Origin of Species* after collecting all of his data but before preparing his 1865 lectures on them. According to this timeline, *Origin of Species* could have neither motivated nor guided his experiments, but it could have influenced his interpretations. Fisher’s (1936) paper “Has Mendel’s Work been Rediscovered?” is mostly remembered for inciting the Mendel–Fisher controversy, though it is a considerably more expansive paper. Among the Fisher’s principal points is his assertion that Darwin influenced Mendel. In reference to a paragraph wherein Mendel rejected the notion that “conditions of life” cause new heritable variation, Fisher remarked, “The reflection of Darwin’s thought is unmistakable….” (p. 134).

Fairbanks and Rytting (2001) published Mendel’s annotations in his copy of *Origin of Species*. They found that the term “Lebensbedingungen” (or its alternatives “Lebens-Bedingungen” and “Lebens Bedingungen”) appear in three of the passages Mendel annotated, each translated from Darwin’s “conditions of life”, which Fairbanks and Abbott (2016) characterised as “a quintessentially Darwinian phrase, appearing 107 times in *Origin of Species*” (p. 404). Mendel used “Lebensbedingungen” twice in his classic paper, both times in the paragraph Fisher emphasised as reflecting Darwin’s thought. This word alone provides evidence supporting Fisher’s assertion, but was only the beginning of such evidence.

Based on the premise that Mendel was reading *Origin of Species* while writing his manuscript, Abbott and Fairbanks (2016) published a new “Darwinised” English translation of Mendel’s classic paper. They compared Mendel’s German text with his German translation of *Origin of Species*, and identified words and phrases in both that were the same, or from the same root. They then employed Darwin’s original English as much as possible in the translation to provide a distinct nineteenth-century tone. Words from passages Mendel annotated in his German translation were conspicuously more frequent near the end of his paper.

At least two, not mutually exclusive, possibilities explain this observation. First, Mendel may have read *Origin of Species* after he had drafted much of his manuscript and therefore these passages influenced the final portion of it. Second, the final two sections of Mendel’s paper deal with theoretical interpretation, and are more amenable to discussions of topics Darwin addressed.

**Fig. 1:**
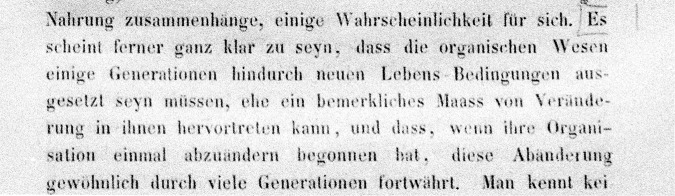
Mendel’s annotation on the first page of the first chapter in his German translation of *Origin of Species*. In Darwin’s original English, the entire sentence reads: “It seems pretty clear that organic beings must be exposed during several generations to the new conditions of life to cause any appreciable amount of variation; and that when the organisation has once begun to vary, it generally continues to vary for many generations”. Note the term “Lebens-Bedingungen”, corresponding to Darwin’s “conditions of life”.

Fairbanks and Abbott (2016) discovered that a significant number of these words appear for the first time in Mendel’s paper in the same paragraph pointed out by Fisher (1936). In this paragraph, Mendel wrote, “The opinion has often been expressed that the stability of a species has been disrupted to a high degree or utterly broken through cultivation” (Abbott and Fairbanks, 2016, p. 418). This is probably a veiled reference to Darwin in response to the first passage Mendel marked in his copy of *Origin of Species* (Fig. 1). It reads, in Darwin’s original English, “It seems pretty clear that organic beings must be exposed during several generations to the new conditions of life to cause any appreciable amount of variation; and that when the organisation has once begun to vary, it generally continues to vary for many generations” (Darwin 1861, p. 7). The “new conditions of life”, in this context, are those under domestication, which, in Darwin’s view, cause a greater degree of inherited variation compared to those in nature.

Mendel clarified in his classic paper how his discoveries were inconsistent with this view: “typical variations must appear if the conditions of life are changed for a species, and it has the ability to adapt to the new conditions. … If the change in the conditions of vegetation were the *sole* cause of variability, then one would be justified in expecting that those domesticated plants cultivated under almost the same conditions for centuries would have acquired *stability*. As is well known, this is not the case…” (italics in original, Abbott and Fairbanks 2016, pp. 418–419).

Here Mendel took a somewhat different approach than Darwin, aligning his view with one Unger had proposed 14 years earlier—that changing conditions of life are not the cause of new hereditary variation: “The endeavour, therefore, to trace the diversities of species to the effect of outward influences, such as the nature of the soils, assuredly misses the true cause” (Unger 1853, p. 93). The notion that changing conditions of life may shape heritable variation was a topic of contentious debate well before Darwin, extending back at least to the eighteenth century (Roberts 1929; Olby 1967). Mendel made it clear that his observations were pertinent to this debate.

Mendel was not the only one in Brno to address this topic. The Natural Science Society in Brno met monthly, and the presentation for January 1865 was by Alexander Makowsky, a botanist and palaeontologist who was Mendel’s friend, research collaborator, and author of a book for which Mendel was a contributing author. Mendel and Makowsky were both teachers at the *Realschule* in Brno, which was the venue for the Natural Science Society’s monthly meetings (Orel 1996). Makowsky’s presentation, “Ueber Darwin’s Theorie der organischen Schöpfung” (“On Darwin’s Theory of Organic Creation”), was a powerful endorsement of Darwin’s *Origin of Species* (Makowsky 1866; Fairbanks and Abbott 2019). Mendel’s presentations would immediately follow Makowsky’s, occupying the next two meetings, in February and March. In his speech, Makowsky addressed Darwin’s “conditions of life” but sided with the view Mendel would also present, downplaying the role of the environment as a cause of new heritable variation.

Given this background, what do we know of Mendel’s views on Darwin specifically, and evolution generally, at the time of his classic paper? The introduction to his 1866 paper contains an often-quoted reference to evolution: “Some courage is certainly required to undertake such an extensive work; nevertheless, it seems to be the only proper means to finally reach resolution of a question regarding the evolutionary history of organic forms, the importance of which must not be underestimated” (Abbott and Fairbanks 2016, p. 407). Later in the paper, he wrote: “This circumstance is especially important for the evolutionary history of plants because constant hybrids acquire the status of *new species*. The truth of this fact has been authenticated by the most preeminent observers and cannot be doubted” (italics in original, Abbott and Fairbanks 2016, p. 420).

In both of these cases, Mendel used the compound term “Entwicklungsgeschichte” (hyphenated in one case), which can be translated as either “evolutionary history” or “developmental history”. “Evolution” (spelled the same in German and English) was not as common a word in either language at the time to denote what became twentieth-century biological evolution. In German, “Entwicklung”, and its derivatives, brought development and evolution together as a unified whole. It is often stated that Darwin never used the word “evolution” in *Origin of Species*, which evades the fact that he employed the past-participle form “evolved” as literally the book’s last word: “… endless forms most beautiful and most wonderful have been, and are being, evolved” (Darwin 1859, p. 490). Heinrich Georg Bronn, the translator of the German version Mendel owned, translated Darwin’s “evolved” as “entwickelt”, from the same root as “Entwicklung”, the word Mendel would have associated more than any other with biological evolution.

#### The enigma of Mendel as evolutionist or creationist

Mendel was a scientist and cleric during a time when the conflict between evolution and religion had reached a feverish pitch, which raises questions regarding his views. Most historians who have examined Mendel’s history conclude that he accepted evolution and viewed his experiments and theory as compatible with Darwin’s (see Iltis, 1966, Olby, 1985; Orel, 1996; Fairbanks and Rytting, 2001, and Klein and Klein, 2013 for detailed discussions). Gliboff (1999) suggested further that Unger’s theory of evolutionary descent strongly influenced Mendel before he learned of Darwin. However, a minority of historians have argued the opposite. Callender (1988) claimed that “Mendel was an opponent of the fundamental principle of evolution itself—that is to say, of *descent with modification*….” (p. 41), and “Mendelism came into being historically as a sophisticated form of the doctrine of Special Creation”, which “stripped of overt references to a Creator, stood in open conflict with the Darwinian conception of evolution” (p. 72).

The most extreme viewpoint is that of Bishop (1996), who wrote, “Mendel’s sole objective in writing his *Pisum* paper, published in 1866, was to contribute to the evolution controversy that had been raging since the publication of Darwin’s the *Origin of Species* in 1859”, and “Mendel was in favour of the orthodox doctrine of special creation” (p. 212). Bishop attempted to show that Mendel initiated his experiments in 1861 (not 1856 as Mendel stated) as a direct reaction to learning of Darwin. This argument is based on the notion proposed by Bateson (1909) and augmented by DiTrocchio (1991) that Mendel’s account of his experiments was fictitious. Orel (1971a) and Fairbanks and Rytting (2001) compiled botanical and historical evidence definitively refuting both the claim that Mendel’s experiments were fictitious and the anachronistic assertion that Darwin’s *Origin of Species* prompted Mendel to initiate them. This evidence augmented Fisher’s (1936) well-researched conclusion that “there can, I believe, be no doubt whatever that his [Mendel’s] report is to be taken entirely literally, and that his experiments were carried out in just the way and in much the order that they are recounted” (p. 132).

In the penultimate paragraph of his 1866 paper, Mendel noted Gärtner’s (1849) view that each species has “fixed limits beyond which it cannot change”, echoing Linnaeus’s words (Aulie 1983). Authors of a number of articles in modern creationist literature and websites have relied on this statement to claim that Mendel supported the fixity of species as a tenet of creationism. The next sentence, however, provides Mendel’s inference that “this view cannot be afforded unconditional validity”. Mendel concluded that Gärtner’s experiments confirmed the “supposition made earlier about the variability of cultivated plants”, which is not an argument that species are fixed, but rather that changing conditions of life under domestication are not “so extremely augmented that species soon lose all stability” (Abbott and Fairbanks 2016, p. 419).

Claims that Mendel was an orthodox proponent of special creation are highly conjectural. Mendel clearly did not fit the prototypical mould of a cleric; nonetheless, available evidence indicates that he remained devoted to his vows. Fragments of undated handwritten notes for two sermons reveal his use of botanical imagery to present deeply pious and heartfelt messages. In these notes, Mendel wrote, “So must the natural and supernatural unite in the realisation of the sanctity of man” (Zumkeller 1971, p. 250*). However, the complete absence of any allusion to the supernatural or to religion in his scientific writings, published and private, indicates that he compartmentalised his religious and scientific views, consistently treating all scientific pursuits with the utmost objectivity.

### Mendel and Darwin after Mendel’s classic paper (1866–84)

Is there any evidence that Darwin read Mendel’s paper, or read about him? This question has been debated for more than 50 years (Vorzimmer 1968), and the short answer is a qualified “no”. Mendel obtained forty offprints of his paper; the fate of only a few is known (Orel 1976, 1996). A rumour purports that an uncut offprint of Mendel’s paper was discovered in Darwin’s collection after his death (for examples see Hennig 2000; Leonard 2005; Fishman 2018), with no credible evidence to support it. It probably arose from the fact that Focke’s (1881) book, *Die Pflanzen-Mischlinge* (*The Plant Hybrids*) was in Darwin’s library, with summaries of Mendel’s experiments, yet the pages of these summaries remain uncut. The fact that Darwin owned this book probably morphed into the rumour that he had an uncut offprint of Mendel’s paper, when in reality he had an uncut reference to it, acquired little more than a year before his death (Fairbanks and Rytting 2001).

#### Mendel’s correspondence with Nägeli

Another question is why there is no evidence that anyone, including Mendel, attempted to inform Darwin of his work. Mendel sent an offprint to Carl Wilhelm von Nägeli, who responded, initiating a collaboration lasting from 1867 through 1873. Nägeli is often criticised for failing to fully grasp Mendel’s 1866 paper, but another oversight is his apparent neglect to inform Darwin of Mendel. Nägeli corresponded with Mendel and Darwin at close to the same time, sending his first letter to Mendel on February 24, 1867 (Darbishire 1906), and his first to Darwin on March 31, 1867 (Burkhardt and Secord 2006). Although most of Nägeli’s correspondence with Darwin has been lost, there is no indication in what remains that he mentioned Mendel, even though his correspondence with both addressed the same plant: *Hieracium* (hawkweed).

Common criticism that Nägeli inadvertently side-tracked Mendel by encouraging him to conduct research on *Hieracium* is not well founded. Mendel fully expected the results he observed in *Hieracium*, for it confirmed his description of constant hybrids in his classic 1866 paper (Van Dijk and Ellis 2016). In Mendel’s mind, *Pisum* hybrids were variable, and *Hieracium* hybrids were constant (Mendel 1870). Moreover, Mendel and Nägeli were far from alone in researching *Hieracium*. In the 1860s and 70s, this genus had become a model for plant evolution in terms of hybridisation and biogeography. Others who were actively researching *Hieracium* at the same time were Anton Kerner von Marilaun, Alexander Makowsky, and Gustav Niessl von Mayendorf, the latter two Mendel’s friends, collaborators, and fellow members of the Natural Science Society in Brno (Iltis 1966).

Nägeli was a well-known proponent of evolution and of Darwin. Mendel’s (1870) paper “Über einige aus künstlicher Befruchtung gewonnenen *Hieracium*-Bastarde” (“On *Hieracium* Hybrids Obtained By Artificial Hybridisation”) referred to Nägeli twice, first without naming him but connecting him with Darwin: “The question of the origin of the numerous and constant intermediate forms has recently acquired no small interest since a famous *Hieracium* specialist has, in the spirit of the Darwinian teaching, defended the view that these forms are to be regarded as [arising] from the transmutation of lost or still-existing species” (Stern and Sherwood, 1966, p. 51). This is Mendel’s only overt mention of Darwin in his publications. Later in the paper, Mendel acknowledged Nägeli by name for sending him plant materials.

#### Mendel’s annotations in Darwin’s “The Variation of Animals and Plants Under Domestication”

Mendel mentioned Darwin three times privately in letters to Nägeli, each instance in 1870 referring to Darwin’s *The Variation of Animals and Plants Under Domestication* (Darwin 1868a, 1868b). Mendel had a German translation of this book (Darwin 1868c, 1868d), and annotated the second volume extensively. Orel (1996) counted only five annotations in Mendel’s copy of the first volume (which is unbound and mostly uncut), compared to 57 in the second (which is cut and bound). To date, Mendel’s annotations in volume 2 have been cited only as a few selected quotations (Orel 1971b, 1996). I have compiled them in full, as numbered entries, in Supplementary Information File 1 (SIF1).

Of the first five annotations (SIF1 entries 1–5), three are in Chapter 15, (On Crossing), one is in Chapter 22 (Causes of Variability), and one is in Chapter 26 (Laws of Variation Continued—Summary). They recount observations that Darwin is unable to explain, but are fully explainable by Mendel’s theory. For example, the second annotated passage recounts a Mendelian dominant-recessive pattern, albeit in non-quantitative terms (in Darwin’s original English): “these colours were never blended, but the offspring bore either pure white or pure yellow blossoms; the former in the larger proportion”. (p. 124, SIF1 entry 2).

The vast majority of annotations are highly clustered (52 of 57) in Chapter 27, (Provisional Hypothesis of Pangenesis), and are focused on three themes: (1) pangenesis itself, (2) the relative contributions of female and male parents during fertilisation, and (3) the influence of changing conditions of life on inherited variation. Mendel addressed these latter two themes in his classic paper (Mendel 1866).

Mendel’s annotations in Chapter 27 tend to highlight Darwin’s uncertainties regarding pangenesis, as well as inferences by Darwin that are inconsistent with Mendel’s experimental observations. The first marked passage in this chapter reads, “As Whewell, the historian of the inductive sciences, remarks:—‘Hypotheses may often be of service to science, when they involve a certain portion of incompleteness, and even of error’” (p. 357, SIF1 entry 6). Later Mendel marked, “The existence of free gemmules is a gratuitous assumption...” (p. 378, SIF1 entry 21). In both of these passages, Darwin admits that his hypothesis of pangenesis is highly speculative with little evidentiary support, and Mendel’s annotations, both here and later, reveal his agreement.

**Fig. 2:**
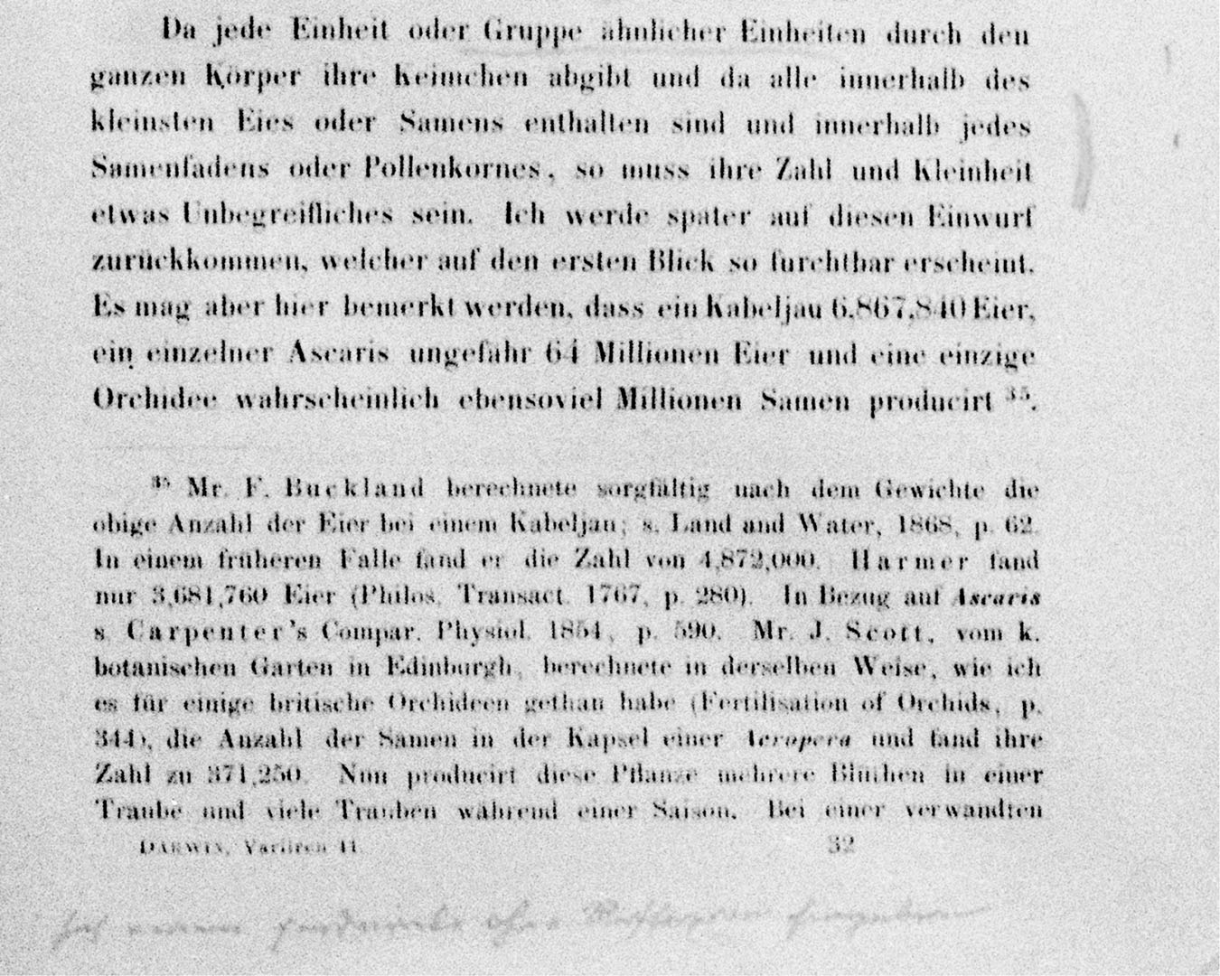
Mendel’s annotations on page 497 of his German translation of Darwin’s *The Variation of Animals and Plants Under Domestication*. The sentence noted by Mendel states (in Darwin’s original English, underlining corresponding to Mendel’s underlined words): “As each unit, or group of similar units throughout the body, casts off its gemmules, and as all are contained within the smallest egg or seed, and within each spermatozoon or pollen grain, their number and minuteness must be something inconceivable”. At the bottom of the page, Mendel wrote (in English translation) “to indulge in an impression without reflection”*.

The part of the book that apparently caught Mendel’s attention more than any other is one he marked with double vertical lines in the margin, next to it a large exclamation point, some underlining of the words, and a handwritten note at the bottom of the page (Fig. 2, SIF1, entries 23 and 24). Darwin’s sentence reads, “As each unit, or group of similar units throughout the body, casts off its gemmules, and as all are contained within the smallest egg or seed, and within each spermatozoon or pollen grain, their number and minuteness must be something inconceivable” (p. 379, SIF1 entry 23). Mendel’s handwritten note is, “sich einem Eindrucke ohne Reflexion hingeben” (“to indulge in an impression without reflection”*). Here, Mendel reveals his scepticism of Darwin’s unsupported view of innumerable gemmules being “cast off” by units within the body and accumulating in the reproductive cells.

A marked passage near the end of the chapter states, “So, also, hybridised plants can be multiplied to any extent by buds, but are continually liable to reversion by seed,—that is, to the loss of their hybrid or intermediate character. I can offer no satisfactory explanation of this fact” (p. 396, SIF1 entry 36). Mendel provided a very satisfactory explanation of such reversion in his classic paper (1866), observing that the proportion of homozygotes increases with repeated self-fertilisation, a phenomenon he demonstrated both experimentally and mathematically, whereas propagation by buds is vegetative, not sexual. Mendel’s explanation, of course, contradicts pangenesis. A brief perusal of the 52 passages Mendel marked in this chapter, most of which are inconsistent with his discoveries, makes it abundantly clear why he wrote to Nägeli, “Darwin’s statements concerning hybrids of the genera mentioned in ‘The Variation of Animals and Plants Under Domestication’, based on reports of others, need to be corrected in many respects” (Stern and Sherwood 1966, p. 93).

Regarding the relative contributions of inherited material by female and male parents, Darwin asserts that the contributions may or may not be equal, that the male parent must contribute a substantial number of gemmules to the egg for successful fertilisation, and, consequently, fertilisation in plants requires more than one pollen grain. Mendel marked ten passages in which Darwin addresses this topic (SIF1, entries 11, 26, 27, 28, 29, 30, 32, 46, 47, and 53).

**Fig. 3:**
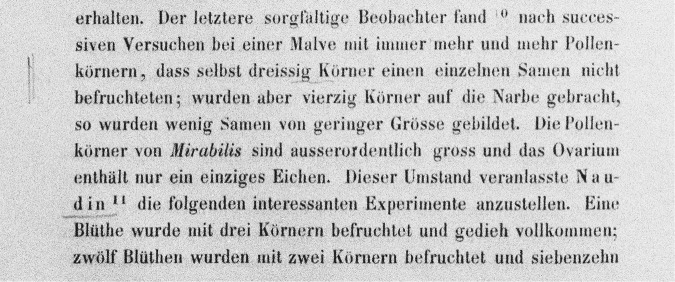
Mendel’s annotations on page 478 of his German translation of Darwin’s *The Variation of Animals and Plants Under Domestication*. The segment marked by Mendel states (in Darwin’s original English, underlining corresponding to Mendel’s underlined words): “even thirty grains did not fertilise a single seed; but when forty grains were applied to the stigma, a few seeds of small size were formed”. Mendel also underlined “Naudin”, who, as explained by Darwin, observed that multiple pollen grains were required for fertilisation in *Mirabilis* (see SIF1 entry 11 for the full English text of this passage). This annotation is especially important because it prompted Mendel, in spite of eye strain, to repeat Naudin’s experiments, recounting them in a letter to Nägeli. Mendel discovered that in repeated cases one pollen grain was, in fact, sufficient for fertilisation, consistent with his theory of heredity.

Even though Mendel was suffering from severe eye strain, he wrote to Nägeli, “But one experiment seemed to me to be so important that I could not bring myself to postpone it to some later date. It concerns the opinion of Naudin and Darwin that a single pollen grain does not suffice for fertilisation of the ovule” (Stern and Sherwood 1966, p. 92). Mendel wrote to Nägeli that he had hand-fertilised flowers of *Mirabilis jalappa* with single pollen grains, and, contradictory to Darwin’s claim in this passage, obtained “18 well developed seeds and an equal number of plants, of which 10 are already in bloom” (Stern and Sherwood, 1966, p. 92). This comment to Nägeli directly corresponds with Mendel’s annotations on page 478 of his German translation, where Darwin recounts the experiments to which Mendel refers in his letter to Nägeli (Fig. 3, SIF1, entry 11).

Mendel had previously addressed this topic in his classic 1866 paper: “According to the view of famous physiologists, in phanerogams, for the purpose of reproduction, one germ cell and one pollen cell unite into a single cell that is able to develop into an independent organism” (Abbott and Fairbanks 2016, p. 420). The “famous physiologists” undoubtedly include Unger, who had been embroiled in a dispute with Eduard Fenzl (Mendel’s other botany professor) when Mendel studied with them. Unger argued that a single pollen cell fertilises a single egg cell ensuring equal parental contributions to each progeny plant. Fenzl claimed that fertilising pollen does not contribute genetic material but serves merely to nourish the egg (Olby, 1985; Orel, 1996; Klein and Klein, 2013). Mendel’s observations were fully consistent with Unger’s assertion, and Mendel included a lengthy footnote supporting Unger’s view, and contradicting Fenzl’s, without naming either (Abbott and Fairbanks 2016, p. 420).

Mendel mentioned Darwin again in his ninth letter to Nägeli: “Darwin and Virchow have pointed to the high degree of independence that is typical for individual characters and whole groups of characters in animals and plants. The behaviour of plant hybrids indisputably furnishes an important proof of the correctness of this point of view” (Stern and Sherwood 1966, p. 96). Although Mendel’s two earlier references to Darwin are criticisms regarding specific topics, this one objectively praises the accuracy of Darwin’s insights.

#### Mendel’s Darwinian statement to Nägeli

Toward the end of Mendel’s tenth letter to Nägeli (November 18, 1873) is perhaps the most Darwinian passage among his existing writings. Here Mendel inferred that several wild species of *Hieracium*, which naturally self-pollinate but occasionally produce hybrids in nature, would ultimately suffer extinction, because if the hybrids, “were repeated too often or became permanent, [they] would finally result in the disappearances of the species involved, while one or another of the more happily organized progeny, better adapted to the prevailing telluric and cosmic conditions, might take up the struggle for existence successfully and continue it for a long stretch of time, until finally the same fate overtook it” (Stern and Sherwood 1966, p. 102). The term “struggle for existence” is, of course, characteristically Darwinian, and the words Mendel used, “Kampf ums Dasein” are the same as in his German translations of *Origin of Species* and *The Variations of Animals and Plants Under Domestication* for Darwin’s phrase “struggle for existence”. Commenting on this passage, Iltis (1966) exclaimed, “the clericalists who stigmatised Mendel the liberal as a Darwinist were not so far wrong!” (p. 204).

The gap between Mendel’s ninth and tenth letters to Nägeli exceeded three years. Mendel noted in his tenth letter that he would send his remaining *Hieracium* hybrids to Nägeli. From that time forward, Mendel dramatically curtailed his plant hybridisation research. Nägeli would twice again write to Mendel, but there is no record that Mendel responded (Iltis 1966). Mendel had managed to conduct research in addition to administrative duties from the time he was elected abbot in 1868, but those duties grown more cumbersome. He increasingly faced hostilities from his superiors, in part due to his dedication to science and in part due to his obstinance in defying demands from church and governmental authorities. These hostilities would escalate in the coming years, leaving him consumed, bitter, and distrustful until his death, January 6, 1884.

### Summary and inferences

We can view Mendel’s connection with Darwin specifically, and evolution generally, from two perspectives: first, the intellectual environments that contextualised his scientific pursuits, and second, his own point of view through his writings and book annotations. In view of the first perspective, Mendel was immersed in intellectual environments highly favourable to evolution throughout all of his scientifically productive years, and to Darwin from 1863 onward (Gliboff 1999). In view of the second perspective, Mendel’s writings reveal the influence of these environments, particularly of Darwin, Unger, and Nägeli.

There is no record of Mendel rejecting Darwin’s theory of evolution by natural selection in any of his existing writings. In fact, as previously noted, Mendel proposed a Darwinian natural-selection scenario invoking the “struggle for existence”. His concerns regarding Darwin were not general, rather focused on three specific themes: Darwin’s provisional hypothesis of pangenesis, the relative contributions of female and male parents at fertilisation, and whether changing conditions of life influence inherited variation. For all three of these themes, Mendel based his views on his experimental observations.

Those who were Mendel’s confidants, who lived to the time of the rediscovery to share reminiscences, recalled Mendel’s dispassionate approach to Darwin, consistent with his existing writings. For example, Iltis (1966) interviewed Mendel’s collaborator, Gustav Niessl von Mayendorf, who, in Iltis’s words, “relates that Mendel, who was greatly interested in the idea of evolution, was far from being an adversary of the Darwinian theory, but always when Darwin’s name came up, he said that the theory was inadequate, that something was lacking” (p. 103). Mendel’s nephew, Ferdinand Schindler, a physician who during his schooling lived near Mendel and with his brothers visited him often, offered a similar recollection in a 1902 letter to William Bateson. Apologising for any grammatical errors, Schindler wrote the letter in English, quoted here exactly as in the original:

The died abbot Mendel was a man of *liberal* principles …. He readed with the greatest interest Darwin’s works in the German translation and admired his genius, though he did not agree to all principles of this immortal natural philosopher. But it can be, that my uncle in the latter part of his life, retired from scientical evolutionary questions, because he had many clerical enemies. He said often to us nephews, that we shall find at his heritage, papers for publication, that he could not publish during his life. But we did not receive anything from the cloister, not even a thing for remembrance.

(Coleman 1967, p. 10)

Schindler’s recollection of manuscripts Mendel delayed publishing for fear of “clerical enemies” may shed light on a glaring question: why did Mendel in his publications apparently include veiled references to Darwin, Unger, and Nägeli without naming them? All three were famous at the time for their evolutionary views, Darwin and Unger suffering public vilification from prominent members of the clergy, the latter in a forum Mendel witnessed. The contextual wording in Mendel’s papers is such that the name omissions are not blatant. Fellow naturalists reading Mendel’s papers would have known to whom he was referring, yet the unnamed individuals would not be obvious to someone unfamiliar with the relevant science. This may have been Mendel’s approach to acknowledging evolutionary experts in a way that would not be obvious to his “clerical enemies”. Nonetheless, as is the case all too often, we can only speculate here, given the absence of direct substantiation.

When the existing evidence is considered as a whole, the image that emerges of Mendel is one of a meticulous scientist who addressed evolutionary questions, was well-versed in the evolutionary literature of his day, was willing to privately point out errors on specific topics in Darwin’s books yet commend him on others, and whose overall research on plant hybridisation had evolutionary implications aligned with those of his predecessors and contemporaries. Mendel’s words and annotations portray neither the fervent advocacy of Darwin espoused by several of his closest collaborators, nor the vitriolic refutation of evolution flaunted by some of his fellow clergymen. As Hartl and Fairbanks (2007) put it, “above all Mendel’s paper appears to reflect the author’s simplicity, modesty, and guilelessness” (p. 975). These attributes extend well beyond Mendel’s classic paper; they pervade the whole of his writings and accord with the recollections of those who knew him.

## Supplementary information

Mendel’s Annotations in His German Translation of Darwin’s (1868) The Variation of Animals and Plants Under Domestication, v. 2

## Footnotes

1 For city names, I use the Anglicised name when possible (e.g. Vienna not Wien, Prague not Praha). A number of cities pertinent to Mendel had both Czech and German names in his day, which have not been Anglicised, and they retain their Czech names today. For these, I cite the current Czech name at first use, with the German name in parentheses, and use only the Czech name in all subsequent instances, e.g. Brno (Brünn).

2 English translations are from the cited source, except those indicated by an asterisk (*), which are original translations from the original German with the generous assistance of Scott Abbott, Frank Hailer, and Brian Charlesworth.
