# Supplementary material for: Mendel and Darwin: untangling a persistent enigma
Source: Heredity (Edinb). 2019 Dec 17;124(2):263–73. doi: 10.1038/s41437-019-0289-9 (PMC6972880; doi:10.1038/s41437-019-0289-9)
Supplement: Supplementary file 1 — Mendel’s Annotations in His German Translation of Darwin’s (1868) The Variation of Animals and Plants Under Domestication, v. 2 [file 41437_2019_289_MOESM1_ESM.pdf]

Mendel's Annotations in His German Translation of Darwin's (1868)
The Variation of Animals and Plants Under Domestication, v. 2

Supplementary File to

Mendel and Darwin: Untangling a Persistent Enigma

by Daniel J. Fairbanks

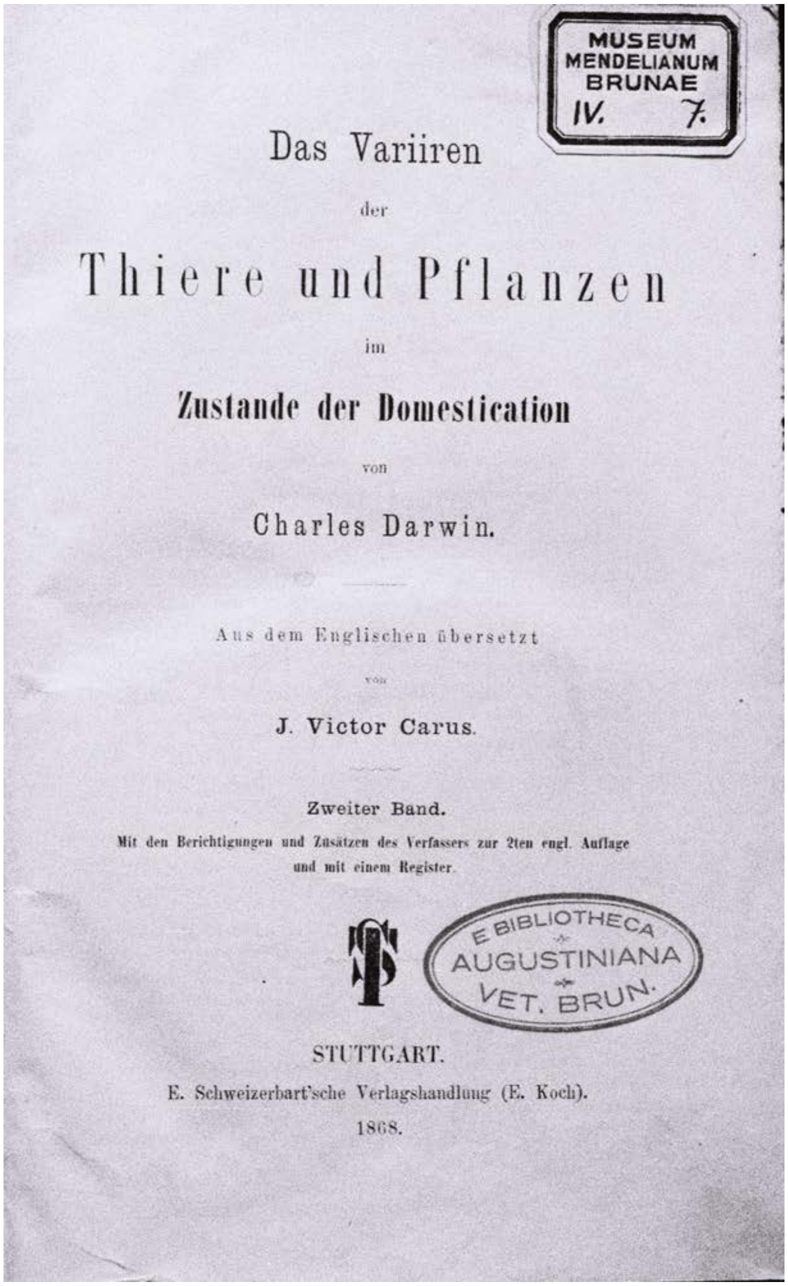
MUSEUM
MENDELIANUM
BRUNAE
IV. 7.

Das Variiren

der

Thiere und Pflanzen

im

Zustande der Domestication

von

Charles Darwin.

Aus dem Englischen übersetzt

von

J. Victor Carus.

Zweiter Band.

Mit den Berichtigungen und Zusätzen des Verfassers zur 2ten engl. Auflage
und mit einem Register.

E BIBLIOTHECA
AUGUSTINIANA
VET. BRUN.

STUTTGART.
E. Schweizerbart'sche Verlagshandlung (E. Koch).
1868.

Gregor Mendel owned both volumes of *The Variations of Animals and Plants Under Domestication* by Charles Darwin (1868) in German translation by Julius Victor Carus. The first volume consists of unbound fascicles, with some pages uncut. According to Orel (1996, pp. 193–194), Mendel made only five annotations in the first volume, whereas he made 57 in the second volume, which is fully cut and bound. My own count also totals 57, although the number could be higher, depending on how one counts adjacent annotations in the same paragraph, on the same page, or spanning page breaks. Given that two of Mendel's handwritten notes run off the page edges, Mendel probably hand-cut the unbound fascicles and annotated the pages before this volume was bound, and the binder re-cut the pages at the time of binding (see entries 18 and 44).

This appendix contains all 57 annotations, which I personally photographed with black-and-white film in 1995. Dr. Anna Matalová, who, at the time, was Head of the Mendelianum of the Moravian Museum in Brno, and curator of the book, extended to me the courtesy and permission to make these photographs. To her I express my gratitude and acknowledgement for this privilege.

I have digitally adjusted brightness and contrast of the photographs to make the annotations as visible as possible without altering the readability of the printed text. Mendel's annotations are often faded and difficult to discern, so in a few cases I digitally enhanced the annotations, separately from the printed text, and layered them exactly in place to make them more visible relative to the printed text.

The annotations are numbered consecutively with photographs of each. Most consist of vertical lines in the margins, with some underlining and handwritten script. Darwin's original English text is provided after each photograph. Because the German texts in the photographs are from the 1868 German translation by Carus, all discrepancies in translation are a consequence of Carus's translation of Darwin's original English into German.

The full context of these passages can be easily viewed by searching the English text given here at the "Darwin Online" site (http://darwin-online.org.uk) for the 1868 first English edition of *The Variation of Animals and Plants Under Domestication* at http://darwin-online.org.uk/content/frameset?itemID=F877.2&viewtype=text&pageseq=1 (accessed December 5, 2019).

Mendel's annotations begin on page 123 German (page 93 English) in Chapter 15 "On Crossing," and end in Chapter 27 "On the Provisional Hypothesis of Pangenesis." I have not commented on any of them in this appendix, reserving commentary for the accompanying article, future publications, and for authors who wish to consult this appendix as a resource for their own research.

— Daniel J. Fairbanks

Reference: Orel V (1996) *Gregor Mendel: The First Geneticist.* Oxford University Press: Oxford.

Chapter 15: On Crossing

1) Page 123 German single vertical line in margin (in footnote):

Analoge Thatsachen sind bei Pflanzen beobachtet worden. Major Trevor Clarke kreuzte den kleinen glattblättrigen einjährigen Levkoj (*Matthiola*) mit dem Pollen einer grossen rothblühenden rauhblättrigen zweijährigen Rasse, die die Franzosen *Corcardeau* nennen, und das Resultat war, dass die Hälfte der Sämlinge glatte, die andere Hälfte rauhe Blätter hatte; aber keine hatten Blätter in einem intermediären Zustande. Dass die glatten Sämlinge das Product der rauhblättrigen Varietät und nicht zufällig das Resultat der Befruchtung mit dem eigenen Pollen der Mutter waren, zeigte sich durch ihr hohes und kräftiges Wachsthumsvermögen [18]. In den folgenden Generationen, die aus den rauhblättrigen gekreuzten Sämlingen erzogen wurden, erschienen einige glatte Pflanzen zum Zeichen, dass der glatte Character, wenn er auch unfähig war, sich mit den rauhen Blättern zu verbinden oder diese zu modificiren, doch die ganze Zeit in dieser Pflanzenfamilie latent vorhanden war. Die zahlreichen

Page 93 English:
Analogous facts have been observed with plants: Major Trevor Clarke crossed the little, glabrous-leaved, annual stock (*Matthiola*), with pollen of a large, red-flowered, rough-leaved, biennial stock, called *cocardeau* by the French, and the result was that half the seedlings had glabrous and the other half rough leaves, but none had leaves in an intermediate state. That the glabrous seedlings were the product of the rough-leaved variety, and not accidentally of the mother-plant's own pollen, was shown by their tall and strong habit of growth. In the succeeding generations raised from the rough-leaved crossed seedlings, some glabrous plants appeared, showing that the glabrous character, though incapable of blending with and modifying the rough leaves, was all the time latent in this family of plants.

2) Page 124 German double lines in margin:

Färbung, sehr nahe denen der väterlichen Pflanze gleich. Gärtner kreuzte viele weiss- und gelbblühende Species und Varietäten von *Verbascum* und diese Färbungen wurden nie verschmolzen, sondern die Nachkommen trugen entweder rein weisse oder reingelbe Blüthen und zwar die ersteren in einem grösseren Verhältniss [19]. Wie mir Dr. Herbert mit-

Page 93 English:
Gärtner crossed many white and yellow-flowered species and varieties of *Verbascum*; and these colours were never blended, but the offspring bore either pure white or pure yellow blossoms; the former in the larger proportion.

3) Page 125 German, double vertical lines in margin:

characteristischen Eigenschaft einer der elterlichen Formen trägt. Bei Bastarden und Mischlingen ereignet es sich häufig oder selbst allgemein, dass ein Theil des Körpers mehr oder weniger nahe dem einen Erzeuger und ein anderer Theil dem andern Erzeuger ähnlich wird; und hier kommt wieder ein gewisser Widerstand gegen die Verschmelzung oder was auf dasselbe hinauskommt, irgend eine gegenseitige Verwandtschaft zwischen den organischen Atomen derselben Natur, wie es scheint, ins Spiel; denn sonst würden alle Theile des Körpers in gleicher Weise im Character intermediär sein. So ferner muss, wenn die Nachkommen

Page 94 English:
With hybrids and mongrels it frequently or even generally happens that one part of the body resembles more or less closely one parent and another part the other parent; and here again some resistance to fusion, or, what comes to the same thing, some mutual affinity between the organic atoms of the same nature, apparently comes into play, for otherwise all parts of the body would be equally intermediate in character.

Chapter 22: Causes of Variability

4) Page 357 German single vertical line in margin:

in einer sichtbaren Weise. Und wir wissen nach den Angaben von Gärtner und Wichura, dass, wenn ein Bastard als Vater benutzt und mit einer reinen Species gekreuzt wird, er den Nachkommen einen bedeutenderen Grad von Variabilität mittheilt, als es derselbe Bastard thut, wenn er als Mutter benutzt wird. Endlich ist es sicher, dass Variabilität durch beide Sexualelemente überliefert werden kann, mag sie ursprünglich in ihnen angeregt sein oder nicht; denn Kölreuter und Gärtner [53] fanden, dass wenn zwei Species, wenn nur eine von ihnen variabel ist, gekreuzt wurden, die Nachkommen variabel wurden.

Page 270 English:
…and we know from Gärtner's and Wichura's statements that a hybrid used as the father and crossed with a pure species gives a greater degree of variability to the offspring, than does the same hybrid when used as the mother. Lastly, it is certain that variability may be transmitted through either sexual element, whether or not originally excited in them, for Kölreuter and Gärtner found that when two species were crossed, if either one was variable, the offspring were rendered variable.

(Note: In the German translation, this sentence begins with "*Und*" [And], whereas in Darwin's original English the corresponding word "and" begins a clause in the sentence, and is thus lower-case, preceded here by an ellipse to indicate that fact.)

Chapter 26: Laws of Variation, continued — Summary

5) Page 458 German, single vertical line in margin:

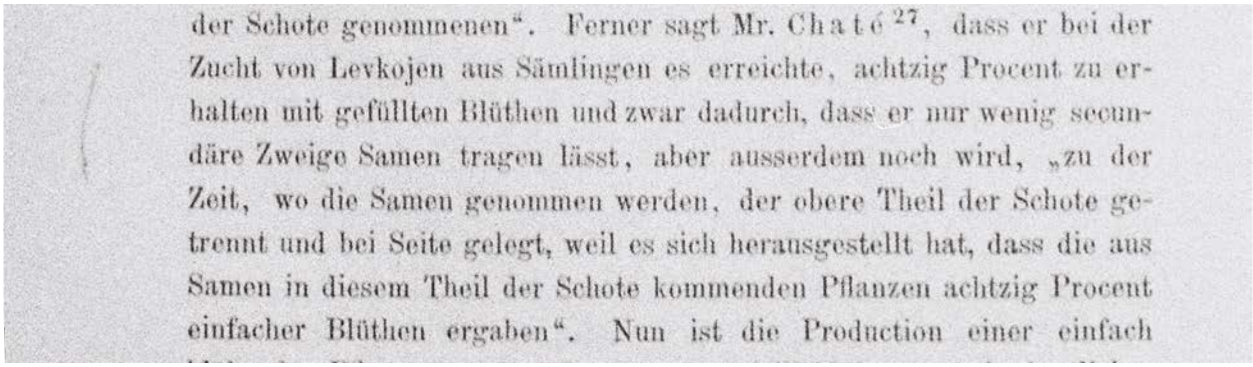
der Schote genommenen". Ferner sagt Mr. Chaté[27], dass er bei der Zucht von Levkojen aus Sämlingen es erreichte, achtzig Procent zu erhalten mit gefüllten Blüthen und zwar dadurch, dass er nur wenig secundäre Zweige Samen tragen lässt, aber ausserdem noch wird, „zu der Zeit, wo die Samen genommen werden, der obere Theil der Schote getrennt und bei Seite gelegt, weil es sich herausgestellt hat, dass die aus Samen in diesem Theil der Schote kommenden Pflanzen achtzig Procent einfacher Blüthen ergaben". Nun ist die Production einer einfach

Page 347–348 English:
Again M. Chaté says says that in raising seedling stocks [genus *Matthiola*] he succeeds in getting eighty per cent. to bear double flowers, by leaving only a few of the secondary branches to seed; but in addition to this, "at the time of extracting the seeds, the upper portion of the pod is separated and placed aside, because it has been ascertained that the plants coming from the seeds situated in this portion of the pod, give eighty per cent. of single flowers."

(Note: The period following "per cent." in in this sentence is in Darwin's original English and indicates an abbreviation for "*per centum*." The translator added the word "Levkojen"

Chapter 27: Provisional Hypothesis of Pangenesis

6) Page 471 German, single vertical line in margin:

[illegible]
same Ursache verbunden, zerstreut vorliegen, zusammengebracht wird. Wie Whewell, der Geschichtsschreiber der inductiven Wissenschaften, bemerkt: „Hypothesen können der Wissenschaft oft von Nutzen sein, wenn sie auch einen gewissen Theil Unvollständigkeit und selbst Irrthum involviren." Unter diesem Gesichts-
[illegible]

Page 357 English:
As Whewell, the historian of the inductive sciences, remarks:—"Hypotheses may often be of service to science, when they involve a certain portion of incompleteness, and even of error."

7) Page 476 German, single vertical line in margin, "Eichen und einer Knopse" ("ovule and a bud") underlined:

der Entwickelung die engste Übereinstimmung besteht. Die Fähigkeit von dem männlichen Element befruchtet zu werden, scheint der Hauptunterschied zwischen einem Eichen und einer Knospe zu sein und diese Fähigkeit tritt nicht unabänderlich in Thätigkeit, wie in den Fällen parthogenetischer Fortpflanzung. Wir werden hier na-
[illegible]

Page 362 English:
The capacity of fertilisation by the male element seems to be the chief distinction between an <u>ovule and a bud</u>; and this capacity is not invariably brought into action, as in the cases of parthenogenetic reproduction. [Underlining by Mendel.]

8) Page 476–477 German, double vertical lines in margin, "Concurrenz" ("concurrence") underlined:

[illegible]
Denn wie ich in einem früheren Capitel gezeigt habe, scheint die Structur jedes Organismus speciell für die Concurrenz zweier Individuen mindestens gelegentlich besonders angepasst zu sein in nahezu derselben Art und Weise. Wie von den Naturforschern zu-

Page 362 English:
…as I have shown in a former chapter, the structure of every organism appears to be especially adapted for the <u>concurrence</u>, at least occasionally, of two individuals. [Underlining by Mendel.]

(Note: Here the translator began the sentence with what Darwin wrote as a clause preceded by another clause in his original English, hence the English is quoted here beginning with a lower-case letter preceded by an ellipse. The sentence spans a page break in German, so the full sentence from both pages is given here, even though Mendel's annotation is only on page 476.)

9) Page 477 German, two successive single vertical lines in margin, "der Keim" ("the germ") underlined:

> gehen.
>
> Warum der Keim, welcher vor der Befruchtung einen gewissen Betrag von Entwickelung erleidet, fortzuschreiten aufhört und abstirbt, wenn er nicht von dem männlichen Element berührt wird und warum umgekehrt das männliche Element, welches fähig ist, selbst vier oder fünf Jahre innerhalb des Samenbehälters eines weiblichen Insectes lebendig zu bleiben, gleichfalls abstirbt, wenn es nicht auf den Keim wirkt oder sich mit ihm verbindet, sind Fragen, welche mit keiner Sicherheit beantwortet werden können. Es ist indess

Page 363 English:
Why <u>the germ</u>, which before impregnation undergoes a certain amount of development, ceases to progress and perishes, unless it be acted on by the male element; and why conversely the male element, which is enabled to keep alive for even four or five years within the spermatheca of a female insect, likewise perishes, unless it acts on or unites with the germ, are questions which cannot be answered with any certainty. [Underlining by Mendel.]

10) Page 478 German, double vertical lines in margin:

> Verbindung gebracht werden, einfach weil sie zu wenig Bildungsmasse enthalten zu einer unabhängigen Existenz und Entwickelung; denn sicher weichen sie in gewöhnlichen Fällen in ihrem Vermögen, dem Embryo einen gewissen Character zu geben, nicht ab. Diese

Page 363 English:
It is, however, possible that both sexual elements perish, unless brought into union, simply from including too little formative matter for independent existence and development; for certainly they do not in ordinary cases differ in their power of giving character to the embryo.

11) Page 478–479 German, double line in margin, “dreissig Korner” (“thirty grains”) underlined, “Naudin” underlined:

erhalten. Der letztere sorgfältige Beobachter fand [10] nach successiven Versuchen bei einer Malve mit immer mehr und mehr Pollenkörnern, dass selbst dreissig Körner einen einzelnen Samen nicht befruchteten; wurden aber vierzig Körner auf die Narbe gebracht, so wurden wenig Samen von geringer Grösse gebildet. Die Pollenkörner von ***Mirabilis*** sind ausserordentlich gross und das Ovarium enthält nur ein einziges Eichen. Dieser Umstand veranlasste Naudin [11] die folgenden interessanten Experimente anzustellen. Eine Blüthe wurde mit drei Körnern befruchtet und gedieh vollkommen; zwölf Blüthen wurden mit zwei Körnern befruchtet und siebenzehn

Blüthen mit einem einzelnen Korn; und von diesen reifte nur eine einzige in jeder Partie ihren Samen; und es verdient besondere Erwähnung, dass die aus diesen beiden Sämlingen erzogenen Pflanzen niemals ihre gehörigen Dimensionen erreichten, und Blüthen von merkwürdig geringer Grösse trugen. Aus diesen Thatsachen

Page 363–364 English:
This last careful observer found, after making successive trials on a Malva with more and more pollen-grains, that even thirty grains did not fertilise a single seed; but when forty grains were applied to the stigma, a few seeds of small size were formed. The pollen-grains of *Mirabilis* are extraordinarily large, and the ovarium contains only a single ovule; and these circumstances led Naudin to make the following interesting experiments: a flower was fertilised by three grains and succeeded perfectly; twelve flowers were fertilised by two grains, and seventeen flowers by a single grain, and of these one flower alone in each lot perfected its seed; and it deserves especial notice that the plants produced by these two seeds never attained their proper dimensions, and bore flowers of remarkably small size. [Underlining by Mendel.]

12) Page 480 German, single vertical line in margin, "Knopsen" ("buds") underlined:

einer inoculirten Krankheit ansehen. Die Möglichkeit, bastardirte Knospen durch die Verbindung zweier distincter pflanzlicher Gewebe zu produciren, ist eine bedeutungsvolle Thatsache, da sie zeigt, dass geschlechtliche oder ungeschlechtliche Fortpflanzung wesentlich identisch sind; denn das Vermögen, in den Nachkommen die Charactere der beiden Eltern zu combiniren, ist die auffallendste von allen Functionen der sexuellen Fortpflanzung.

Pages 364-365 English:
Should it ever be proved that hybridised buds can be formed by the union of two distinct vegetative tissues, the essential identity of sexual reproduction would be shown in the most interesting manner; for the power of combining in the offspring the characters of both parents, is the most striking of all the functions of sexual generation. [Underlining by Mendel.]

13) Page 485 German, single vertical line in margin, "dem unabhangigen" ("the independent") underlined:

auf der andern Seite repräsentirt wäre."

Viele Thatsachen unterstützen diese Ansicht von dem unabhängigen Leben jedes kleinsten Elements des Körpers. Virchow

Page 369 English:
Many facts support this view of the independent life of each minute element of the body. [Underlining by Mendel.]

14) Page 492 German, double vertical lines in margin, "zu ihrer Aggregation" ("to their aggregation") and "Sexualelementen" ("sexual elements") underlined:

sondern während aller Entwicklungszustände derselben abgegeben werden. Endlich nehme ich an, dass die Keimchen in ihrem schlummernden Zustande eine gegenseitige Verwandtschaft zu einander haben, welche zu ihrer Aggregation entweder zu Knospen oder zu den Sexualelementen führt. Um genauer zu sprechen, so sind es nicht die reproductiven Elemente, auch nicht die Knospen, welche neue Organismen erzeugen, sondern die Zellen selbst durch den ganzen Körper. Diese Annahmen bilden die provisorische Hypo-

Page 374 English:
Lastly, I assume that the gemmules in their dormant state have a mutual affinity for each other, leading to their aggregation either into buds or into the sexual elements. Hence, speaking strictly, it is not the reproductive elements, nor the buds, which generate new organisms, but the cells themselves throughout the body. [Underlining by Mendel.]

15) Page 492 German, in footnote 30, single vertical line in margin:

Hätte Buffon angenommen, dass seine organischen Molecule von jeder einzelnen Einheit durch den ganzen Körper gebildet worden wären, so würden seine und meine Ansicht einander sehr ähnlich sein.

Page 375 English, in footnote:
If Buffon had assumed that his organic molecules had been formed by each separate unit throughout the body, his view and mine would have been closely similar.

16) Page 492 German, in footnote 30, single vertical line in margin:

rationen fertig eingeschlossen seien. Meiner Ansicht zufolge waren die Keime oder Keimchen jedes einzelnen Theiles nicht ursprünglich präformirt, sondern werden beständig in jedem Alter während jeder Generation producirt, wobei einige von früheren Generationen weiter überliefert werden.

Page 375 English:
According to my view, the germs or gemmules of each separate part were not originally preformed, but are continually produced at all ages during each generation, with some handed down from preceding generations.

17) Page 493 German, single vertical line in margin:

chen befruchtet worden sei. Von meinen Keimchen wird angenommen, dass sie sich völlig unabhängig vom Zusammenwirken der beiden Geschlechter von jeder einzelnen Zelle oder Einheit durch den ganzen Körper bilden und dass sie innerhalb der Reproductionsorgane einfach aggregirt werden.

Page 375 English:
My gemmules are supposed to be formed, quite independently of sexual concourse, by each separate cell or unit throughout the body, and to be merely aggregated within the reproductive organs.

18) Page 493 German, faded-pencil script in right margin running off edge of cut page, illegible, “Verwandtschaft” (“affinity”) underlined in printed text:

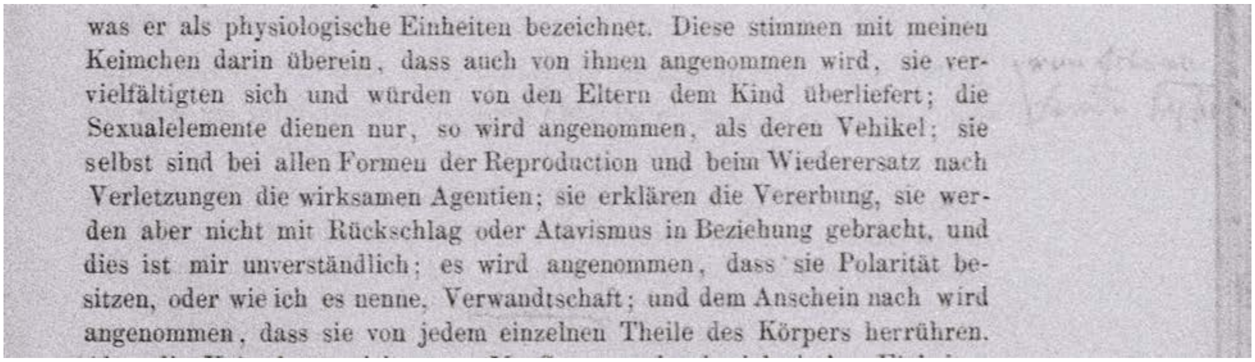

was er als physiologische Einheiten bezeichnet. Diese stimmen mit meinen Keimchen darin überein, dass auch von ihnen angenommen wird, sie vervielfältigten sich und würden von den Eltern dem Kind überliefert; die Sexualelemente dienen nur, so wird angenommen, als deren Vehikel; sie selbst sind bei allen Formen der Reproduction und beim Wiederersatz nach Verletzungen die wirksamen Agentien; sie erklären die Vererbung, sie werden aber nicht mit Rückschlag oder Atavismus in Beziehung gebracht, und dies ist mir unverständlich; es wird angenommen, dass sie Polarität besitzen, oder wie ich es nenne, Verwandtschaft; und dem Anschein nach wird angenommen, dass sie von jedem einzelnen Theile des Körpers herrühren.

Page 375 English text adjacent to script:
These agree with my gemmules in being supposed to multiply and to be transmitted from parent to child; the sexual elements are supposed to serve merely as their vehicles; they are the efficient agents in all the forms of reproduction and in the repairs of injuries; they account for inheritance, but they are not brought to bear on reversion or atavism, and this is unintelligible to me; they are supposed to possess polarity, or, as I call it, affinity; and apparently they are believed to be derived from each separate part of the whole body. [Underlining by Mendel.]

19) Page 493 German, In script at bottom of page “physiol[ogischer] Einheiten = Keimchen” (“physiological units = gemmules”). On the same page, Darwin had equated his “gemmules” with what Herbert Spencer called “physiological units.”

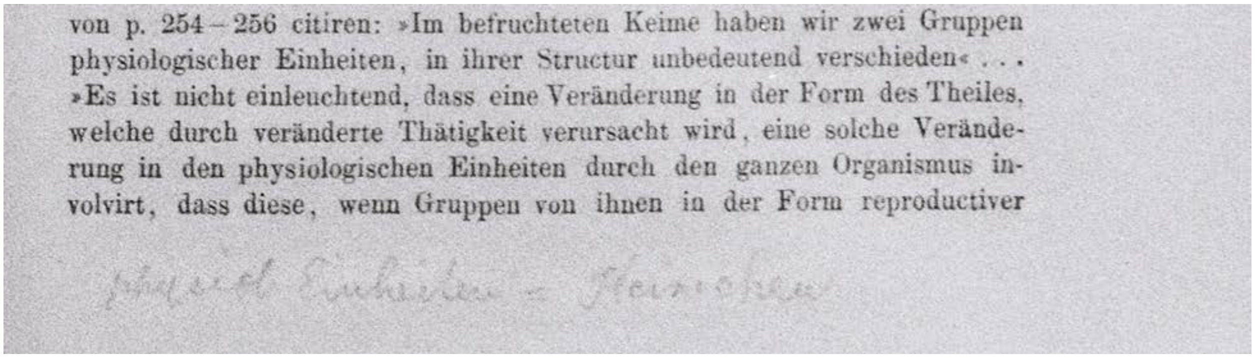

von p. 254–256 citiren: »Im befruchteten Keime haben wir zwei Gruppen physiologischer Einheiten, in ihrer Structur unbedeutend verschieden« . . . »Es ist nicht einleuchtend, dass eine Veränderung in der Form des Theiles, welche durch veränderte Thätigkeit verursacht wird, eine solche Veränderung in den physiologischen Einheiten durch den ganzen Organismus involvirt, dass diese, wenn Gruppen von ihnen in der Form reproductiver

physiol Einheiten = Keimchen

20) Page 496 German, double lines in margin, “Zelle ein freies Keimchen abgibt” (“cell casts off a free gemmule”) and “ähnliche Zelle” (“similar cell”) underlined:

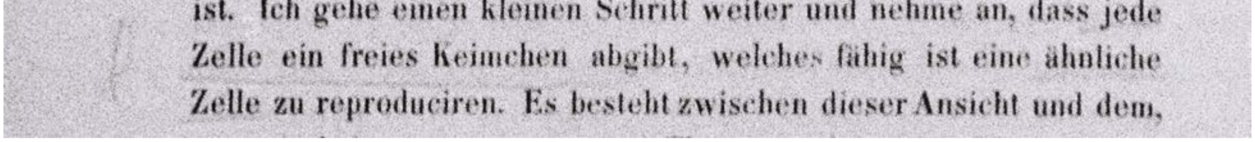

ist. Ich gehe einen kleinen Schritt weiter und nehme an, dass jede Zelle ein freies Keimchen abgibt, welches fähig ist eine ähnliche Zelle zu reproduciren. Es besteht zwischen dieser Ansicht und dem,

Page 377 English:
I go one small step further, and assume that each cell casts off a free gemmule, which is capable of reproducing a similar cell. [Underlining by Mendel.]

21) Page 496 German, single vertical line in margin:

Die Existenz freier Keimchen ist eine willkürliche Annahme, kann aber kaum als sehr unwahrscheinlich betrachtet werden, wenn wir sehen, dass dieselben das Vermögen der Vervielfältigung durch Theilung ihres Inhalts haben. Keimchen weichen von echten Eiern

Page 378 English:
The existence of free gemmules is a gratuitous assumption, yet can hardly be considered as very improbable, seeing that cells have the power of multiplication through the self-division of their contents.

22) Page 497 German, single vertical line in margin:

Das Zurückhalten freier und unentwickelter Keimchen in demselben Körper von früher Jugend bis zum hohen Alter kann unwahrscheinlich erscheinen; wir müssen uns aber daran erinnern, wie lange Samen in der Erde schlummernd liegen und Knospen in der Rinde eines Baumes schlummern können. Ihre Überlieferung von Generation zu Generation kann noch unwahrscheinlicher erscheinen; aber auch hier müssen wir uns erinnern, dass viele rudimentäre und nutzlose Organe durch eine unendliche Zahl von Generationen überliefert worden sind. Wir werden sofort sehen, wie gut die lange fortgesetzte Überlieferung unentwickelter Keimchen viele Thatsachen erklärt.

Pages 378-379 English:
The retention of free and undeveloped gemmules in the same body from early youth to old age may appear improbable, but we should remember how long seeds lie dormant in the earth and buds in the bark of a tree. Their transmission from generation to generation may appear still more improbable; but here again we should remember that many rudimentary and useless organs are transmitted and have been transmitted during an indefinite number of generations. We shall presently see how well the long-continued transmission of undeveloped gemmules explains many facts.

23) Page 497 German, double lines and large exclamation point in margin, faint underlining of "Das jede Einheit oder Gruppe ähnlicher Einheiten" ("As each unit, or group of similar units"):

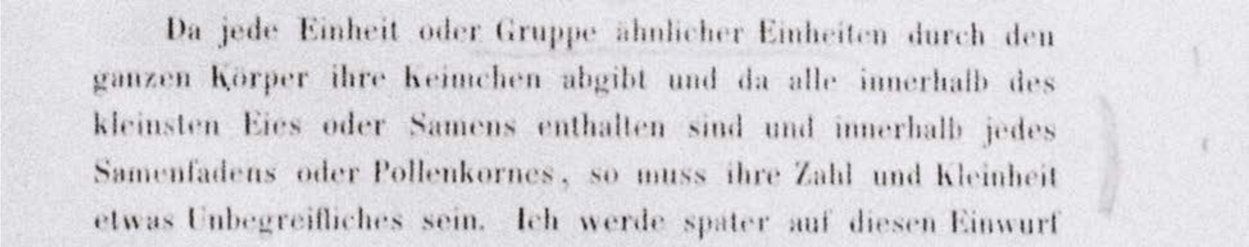
Da jede Einheit oder Gruppe ähnlicher Einheiten durch den ganzen Körper ihre Keimchen abgibt und da alle innerhalb des kleinsten Eies oder Samens enthalten sind und innerhalb jedes Samenfadens oder Pollenkornes, so muss ihre Zahl und Kleinheit etwas Unbegreifliches sein. Ich werde später auf diesen Einwurf

Page 379 English:
As each unit, or group of similar units throughout the body, casts off its gemmules, and as all are contained within the smallest egg or seed, and within each spermatozoon or pollen-grain, their number and minuteness must be something inconceivable. [Underlining by Mendel.]

24) Page 497 German, note in script on the bottom of the page: "sich einem Eindrucke ohne Reflexion hingeben" ("to indulge in an impression without reflection")

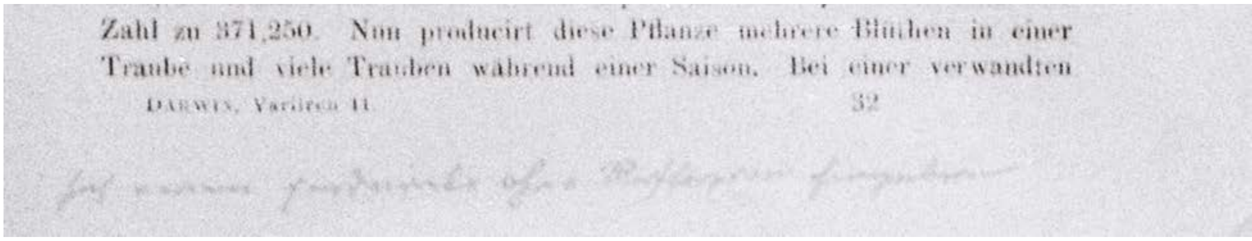
Zahl zu 371,250. Nun producirt diese Pflanze mehrere Blüthen in einer Traube und viele Trauben während einer Saison. Bei einer verwandten

DARWIN, Variiren II. 32

25) Page 499 German, single vertical line in margin:

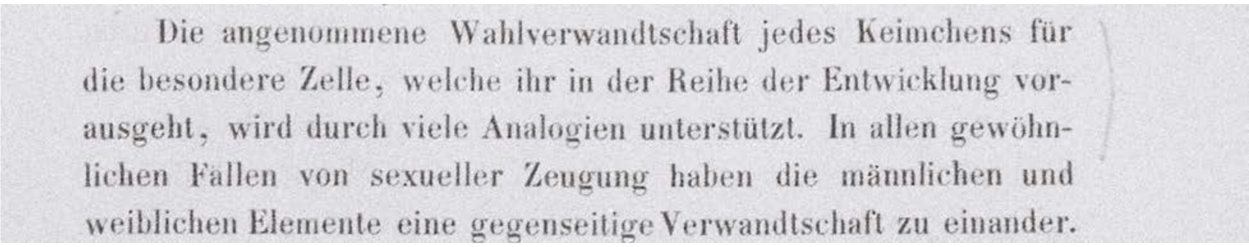
Die angenommene Wahlverwandtschaft jedes Keimchens für die besondere Zelle, welche ihr in der Reihe der Entwicklung vorausgeht, wird durch viele Analogien unterstützt. In allen gewöhnlichen Fällen von sexueller Zeugung haben die männlichen und weiblichen Elemente eine gegenseitige Verwandtschaft zu einander.

Page 380 English:
The assumed elective affinity of each gemmule for that particular cell which precedes it in the order of development is supported by many analogies. In all ordinary cases of sexual reproduction the male and female elements have a mutual affinity for each other:

26) Page 505 German, single vertical line in margin:

dismus. Sie stimmt aber ganz gut mit den meisten der sicher gestellten Thatsachen überein. Aus der Thatsache, dass ein einziger Samenfaden oder ein einziges Pollenkorn zur Befruchtung nicht ausreichend ist, können wir schliessen, dass eine gewisse Zahl von Keimchen, die aus jeder Zelle oder Einheit herrühren, zu der Entwickelung jedes Theiles erforderlich ist. Aus dem Vorkommen

Page 385 English:
We may conclude from the fact of a single spermatozoon or pollen-grain being insufficient for impregnation, that a certain number of gemmules derived from each cell or unit are required for the development of each part.

27) Page 505–506 German, single vertical line in margin on page 506, with exclamation point.

wickelung jedes Theiles erforderlich ist. Aus dem Vorkommen der Parthenogenesis und noch besonders aus dem Falle bei dem Seidenschmetterlinge, wo der Embryo oft theilweise gebildet wird,

können wir auch schliessen, dass das weibliche Element nahezu hinreichende Keimchen aller Arten für eine unabhängige Entwickelung einschliesst, so dass, wenn es sich mit dem männlichen Element vereinigt hat, die Keimchen im Überschuss vorhanden sein müssen. Nun weichen als allgemeine Regel, wenn zwei Species oder Rassen wechselseitig gekreuzt werden, die Nachkommen nicht ab und dies zeigt, dass beide sexualen Elemente ihrer Stärke nach übereinstimmen, der Ansicht entsprechend, dass sie dieselben Keimchen einschliessen. Bastarde und Mischlinge sind gewöhnlich im Character

Page 385 English:
From the occurrence of parthenogenesis, more especially in the case of the silk-moth, in which the embryo is often partially formed, we may also infer that the female element includes nearly sufficient gemmules of all kinds for independent development, so that when united with the male element the gemmules must be superabundant. Now, as a general rule, when two species or races are crossed reciprocally, the offspring do not differ, and this shows that both sexual elements agree in power, in accordance with the view that they include the same gemmules.

28) Page 506 German, single vertical line in margin:

Auch ist dies nach der Annahme, dass die Keimchen in dem befruchteten Keim in überschüssiger Zahl vorhanden sind, und dass diejenigen, die von der einen elterlichen Form herrühren, irgend einen Vortheil in der Zahl, Anziehungskraft oder Lebenskraft über die von der andern elterlichen Form herrührenden besitzen, nicht schwierig zu verstehen. Gekreuzte Formen bieten zuweilen die

Page 386 English:
[Also, this is after] the admission that the gemmules in the fertilised germ are superabundant in number, and that those derived from one parent have some advantage in number, affinity, or vigour over those derived from the other parent.

(Note: Here the translator initiated a new sentence in German, adding the phrase "Auch ist dies nach" ["Also, this is after…"] when translating from a longer sentence by Darwin.)

29) Page 506 German, single vertical line in margin:

Beispielen. In diesen Fällen müssen wir Naudin folgen[43] und annehmen, dass die Essenz oder das Element der beiden Species, welche Ausdrücke ich in die Keimchen übersetzen würde, eine Affinität für ihre eigene Art habe und sich demzufolge in besondere Streifen oder Flecke trenne; und bei der Erörterung im fünfzehn-

Page 386 English:
In these cases we must follow Naudin, and admit that the "essence" or "element" of the two species, which terms I should translate into the gemmules, have an affinity for their own kind, and thus separate themselves into distinct stripes or blotches;

30) Page 506 German, single vertical line in margin:

wechselseitige Affinität anzunehmen. Wenn zwei Formen gekreuzt werden, so findet sich nicht selten, dass die eine bei der Überlieferung der Charactere ein Übergewicht über die andere hat; und diess können wir wieder nur dadurch erklären, dass wir annehmen, die eine Form habe irgend einen Vortheil in der Zahl, Lebenskraft oder

Affinität ihrer Keimchen voraus, mit Ausnahme derjenigen Fälle, wo gewisse Charactere in der einen Form vorhanden sind, in der andern nur latent auftreten. So findet sich z. B. bei allen Tauben

Page 386 English:
When two forms are crossed, one is not rarely found to be prepotent in the transmission of character over the other; and this we can explain only by again assuming that the one form has some advantage in the number, vigour, or affinity of its gemmules, except in those cases, where certain characters are present in the one form and latent in the other.

31) Page 507 German, double lines and exclamation point in margin, "die Annahme" ("the assumption" translated from Darwin's "implies") underlined, "hybridisirte Zellenkeimchen abgeben" ("throw off hybridized cell-gemmules") underlined.

Pflanzen dasselbe Resultat herbeiführen. Der Schluss, dass nur die Reproductionsorgane und nicht die ganze Organisation afficirt ist, stimmt vollkommen mit der unbeeinträchtigten oder selbst vermehrten Fähigkeit bei hybriden Pflanzen überein, sich durch Knospen zu vermehren; denn dies schliesst nach unserer Hypothese die Annahme ein, dass die Zellen der Bastarde hybridisirte Zellenkeimchen abgeben, welche wohl zu Knospen aggregirt werden, aber innerhalb der Reproductionsorgane nicht so aggregirt werden, dass sie Sexualelemente bilden. So produciren in einer ähnlichen Weise viele Pflanzen, wenn

Page 387 English:
The conclusion that the reproductive organs alone are affected, and not the whole organisation, agrees perfectly with the unimpaired or even increased capacity in hybrid plants for propagation by buds; for this implies, according to our hypothesis, that the cells of the hybrids throw off hybridised cell-gemmules, which become aggregated into buds, but fail to become aggregated within the reproductive organs, so as to form the sexual elements. [Underlining by Mendel.]

32) Page 508 German, double vertical lines in margin:

oberen Theil des Bluthenstieles. In Übereinstimmung mit der Hy-
pothese der Pangenesis enthält der Pollen Keimchen, welche von
jedem Theil des Organismus herrühren, sich verbreiten und durch
Theilung vervielfältigen. Es ist daher nicht überraschend, dass Keim-
chen in Pollen, welche von den Theilen in der Nähe der Reproduc-
tionsorgane herrühren, zuweilen im Stande sein sollten, dieselben
Theile in der Mutterpflanze zu afficiren, während sie noch der Ent-
wickelung unterliegen.

Page 387 English:
In accordance with the hypothesis of pangenesis pollen includes gemmules, derived from every part of the organisation, which diffuse themselves and multiply by self-division; hence it is not surprising that gemmules within the pollen, which are derived from the parts near the reproductive organs, should sometimes be able to affect the same parts, whilst still undergoing development, in the mother-plant.

33) Page 508–509 German, single vertical line in margin on page 508:

Da während aller Entwickelungsstadien die Gewebe der Pflan-
zen aus Zellen bestehen, und da man weiss, dass keine neuen Zel-
len zwischen oder unabhängig von präexistirenden Zellen gebildet
werden, so müssen wir schliessen, dass die von fremdem Pollen
herrührenden Keimchen nicht einfach im Contact mit präexistirenden
Zellen entwickelt werden, sondern factisch in die entstehenden Zel-

len der mütterlichen Pflanze eindringen. Dieser Process kann mit
dem gewöhnlichen Befruchtungsact, während welches die Inhalt-

Page 388 English:
As, during all the stages of development, the tissues of plants consist of cells, and as new cells are not known to be formed between, or independently of, pre-existing cells, we must conclude that the gemmules derived from the foreign pollen do not become developed merely in contact with pre-existing cells, but actually penetrate the nascent cells of the mother-plant.

34) Page 509 German, single vertical line in margin:

werden. Wie wir sofort sehen werden, kann man bei allen Orga-
nismen in gleicher Weise sagen, dass die Zellen oder organischen
Einheiten des Embryos während der aufeinander folgenden Stufen
der Entwickelung von den Keimchen derjenigen Zellen befruchtet
werden, welche in der Reihenfolge der Bildung zunächst kommen.

Page 388 English:
With all organisms, as we shall presently see, the cells or organic units of the embryo during the successive stages of development may in like manner be said to be fertilised by the gemmules of the cells, which come next in the order of formation.

35) Page 510 German, double vertical lines in margin:

Der Ausdruck Wachsthum sollte streng genommen auf blosse
Grössenzunahme und der Ausdruck Entwickelung auf Structurver-
änderungen beschränkt werden[44]. Nun sagt man, ein Kind wächst zum

Page 389 English:
The term growth ought strictly to be confined to mere increase of size, and development to change of structure.

36) Page 512 German, single vertical line in margin:

Das Princip der unabhängigen Bildung jedes Theiles, so weit
seine Entwickelung von der Vereinigung der gehörigen Keimchen
mit gewissen in der Entstehung begriffenen Zellen abhängt, in Ver-
bindung mit dem Überschuss von Keimchen, welche von beiden
Eltern herrühren und sich vervielfältigt haben, wirft auch auf eine
sehr verschiedene Gruppe von Thatsachen Licht, welche nach jeder
gewöhnlichen Ansicht von Entwickelung sehr befremdend erscheint.

Pages 390-391 English:
The principle of the independent formation of each part, in so far as its development depends on the union of the proper gemmules with certain nascent cells, together with the superabundance of the gemmules derived from both parents and self-multiplied, throws light on a widely different group of facts, which on any ordinary view of development appears very strange.

37) Page 518 German, single vertical line in margin:

werden; dass aber die Keimchen, welche von denselben Zellen nach
der Modification ausgehen, natürlich unter denselben günstigen Be-
dingungen sich fortwährend vermehren, bis sie endlich hinreichend
zahlreich werden, die alten Keimchen zu überwinden und zu ersetzen.
Es mag eine andere Schwierigkeit hier noch erwähnt werden.

Page 395 English:
…but that the gemmules derived from the same cells after modification, naturally go on increasing under the same favouring conditions, until at last they become sufficiently numerous to overpower and supplant the old gemmules.

38) Page 519 German, single vertical line in margin:

Samen zurück. So können auch verbastardirte Pflanzen in jeder
Ausdehnung durch Knospen vervielfältigt werden, sind aber fort-
während durch Samen dem Rückschlag ausgesetzt, d. h. dem Ver-
luste ihres hybriden oder intermediären Characters. Für diese That-
sachen kann ich keine befriedigende Erklärung geben. Das Folgende

Page 396 English:
So, also, hybridised plants can be multiplied to any extent by buds, but are continually liable to reversion by seed,—that is, to the loss of their hybrid or intermediate character. I can offer no satisfactory explanation of this fact.

39) Page 519 German, Roman numeral “I” in margin with Roman numeral II crossed out below it:

Endlich können wir nach der Hypothese der Pangenesis sehen,
dass die Variabilität mindestens von zwei distincten Gruppen von
Ursachen abhängt, erstens von dem Mangel, dem Überschuss, der
Verschmelzung und Umstellung von Keimchen und von der Wieder-
entwicklung derjenigen, welche lange im ruhenden Zustande gele-
gen haben. In diesen Fällen haben die Keimchen selbst keine Modi-

Page 396 English:
Finally, we can see on the hypothesis of pangenesis that variability depends on at least two distinct groups of causes. Firstly, on the deficiency, superabundance, fusion, and transposition of gemmules, and on the redevelopment of those which have long been dormant.

40) Page 519 German, Roman numeral "II" in margin:

erklären. Zweitens in den Fällen, wo die Organisation durch veränderte Bedingungen, den vermehrten Gebrauch oder Nichtgebrauch von Theilen oder irgend eine andere Ursache modificirt worden ist, werden die von den modificirten Einheiten des Körpers abgeworfenen Keimchen selbst modificirt werden, und werden, wenn sie sich genügend vervielfältigt haben, sich zu neuen und veränderten Gebilden entwickeln.

Page 397 English:
Secondly, in the cases in which the organisation has been modified by changed conditions, the increased use or disuse of parts, or any other cause, the gemmules cast off from the modified units of the body will be themselves modified, and, when sufficiently multiplied, will be developed into new and changed structures.

41) Page 520 German, Single vertical line in margin:

der Vererbung haben [54]. Genau dieselbe Ansicht lässt sich auf die unendlich zahlreichen und verschiedenartigen Einheiten ausdehnen, aus denen der ganze Körper bei einem der höheren Thiere zusammengesetzt ist: und die getrennten Atome sind unsere Keimchen. Wir haben bereits die Vererbung der directen Wirkungen veränderter Bedingungen, vermehrten Gebrauchs oder Nichtgebrauchs von Theilen, und implicite auch das wichtige Princip der Vererbung zu entsprechenden Altern hinreichend erörtert. Diese Gruppen von Thatsachen sind

Page 397 English:
Precisely the same view may be extended to the infinitely numerous and diversified units of which the whole body in one of the higher animals is composed; and the separated atoms are our gemmules. We have already sufficiently discussed the inheritance of the direct effects of changed conditions, and increased use or disuse of parts, and, by implication, the important principle of inheritance at corresponding ages.

42) Page 521 German, single vertical line in margin:

Zweifel die Keimchen selbst zerstört. Auch ist es in keiner Weise unwahrscheinlich; denn obgleich eine ungeheure Zahl von thätigen und lange ruhenden Keimchen in jedem lebenden Wesen diffundirt sind und ernährt werden, so muss es doch irgend eine Grenze für ihre Zahl geben, und es scheint natürlich, dass Keimchen, die von einem geschwächten und nutzlosen Rudimente herrühren, eher zerstört werden, als die, welche von anderen Theilen herrühren, welche noch in voller functioneller Thätigkeit sind.

Pages 397–398 English:
…nor is this in any way improbable, for, though a vast number of active and long-dormant gemmules are diffused and nourished in each living creature, yet there must be some limit to their number; and it appears natural that gemmules derived from an enfeebled and useless rudiment would be more liable to perish than those derived from other parts which are still in full functional activity.

(Note: Here the translator began the sentence with what Darwin wrote as a clause preceded by another clause in his original English, hence the English is quoted here beginning with a lower-case letter preceded by an ellipse.)

43) Page 521 German, single vertical line in margin:

An zweiter Stelle vervielfältigen sich unserer Hypothese zufolge die Keimchen durch Selbsttheilung und werden von Generation zu Generation überliefert, so dass sie eine lange Periode hindurch vorhanden und bereit sein würden, einen wiederholt amputirten Theil zu reproduciren. Nichtsdestoweniger scheint es nach den im zwölf-

Page 398 English:
In the second place, according to our hypothesis, gemmules multiply by self-division and are transmitted from generation to generation; so that during a long period they would be present and ready to reproduce a part which was repeatedly amputated.

44) Pages 521–522 German, Sentence begins on page 521, “Rückschlag” (“reversion”) written in margin on page 522, repeating the same word from the last line of page 521, edge of page 522 cut, removing “R” and left part of “ü”:

Der letzte Gegenstand, der hier erörtert werden muss, nämlich der Rückschlag, beruht auf dem Princip, dass Überlieferung und

Entwickelung, obgleich meist in Verbindung wirksam, distincte Kräfte sind; und die Überlieferung von Keimchen und ihre spätere Entwickelung zeigt uns, wie die Existenz dieser beiden distincten Vermögen möglich ist. Wir sehen diese Verschiedenheit deutlich in den

Page 398 English:
The last subject that need here be discussed, namely Reversion, rests on the principle that transmission and development, though generally acting in conjunction, are distinct powers; and the transmission of gemmules and their subsequent development show us how the existence of these two distinct powers is possible.

45) Page 524 German, single vertical line in margin (long), second vertical line (shorter):

Wie können wir diese Thatsachen erklären? Jede organische Einheit in einem Bastard muss nach der Theorie der Pangenesis eine Menge hybridisirter Keimchen abgeben; denn gekreuzte Pflanzen können leicht und in weitem Umfange durch Knospen fortgepflanzt werden; aber nach derselben Hypothese werden in gleicher Weise schlummernde, von beiden reinen elterlichen Formen herrührende Keimchen vorhanden sein; und da diese letzteren ihren normalen Zustand beibehalten, so ist es wahrscheinlich, dass sie einer bedeutenden Vervielfältigung während der Lebenszeit jedes Bastards fähig sind. In Folge dessen werden die sexuellen Elemente eines Bastards sowohl reine als hybridisirte Keimchen enthalten; und wenn sich zwei Bastarde paaren, so wird die Combination reiner, von dem einen Bastard herrührender Keimchen mit den reinen Keimchen derselben Theile, welche von dem anderen Bastard herrühren, nothwendig zu einem vollständigen Rückschlag im Character führen; und es ist vielleicht keine zu kühne Voraussetzung, dass nicht modificirte und nicht verschlechterte Keimchen von derselben Natur besonders einer Combination geneigt sind. Reine Keimchen in Combination mit hybridisirten Keimchen würden zu einem theilweisen Rückschlag führen. Und endlich hybridisirte von beiden elterlichen Bastarden herrührende Keimchen würden einfach die

Pages 400-401 English:
How can we account for these facts? Each organic unit in a hybrid must throw off, according to the doctrine of pangenesis, an abundance of hybridised gemmules, for crossed plants can be readily and largely propagated by buds; but by the same hypothesis there will likewise be present dormant gemmules derived from both pure parent-forms; and as these latter retain their normal condition, they would, it is probable, be enabled to multiply largely during the lifetime of each hybrid. Consequently the sexual elements of a hybrid will include both pure and hybridised gemmules; and when two hybrids pair, the combination of pure gemmules derived from the one hybrid with the pure gemmules of the same parts derived from the other would necessarily lead to complete reversion of character; and it is, perhaps, not too bold a supposition that unmodified and undeteriorated gemmules of the same nature would be especially apt to combine. Pure gemmules in combination with hybridised gemmules would lead to partial reversion.

46) Page 525 German, Single vertical line in margin:

schmelzen. Wenn daher zwei Thiere mit antagonistischen Characteren gekreuzt werden, so kann es sich wohl treffen, dass eine hinreichende Menge von Keimchen nur in dem Männchen vorhanden ist zur Reproduction seiner eigenthümlichen Charactere und in dem Weibchen nur zur Reproduction seiner eigenthümlichen Charactere; und in diesem Falle können von irgend einem entfernten Vorfahren herrührende Keimchen leicht wieder Übergewicht erlangen und das Wiederauftreten lange verloren gegangener Charactere verursachen.

Page 401 English:
… when two animals with antagonistic characters are crossed, it might well happen that a sufficiency of gemmules in the male alone for the reproduction of his peculiar characters, and in the female alone for the reproduction of her peculiar characters, would not be present; and in this case dormant gemmules derived from some remote progenitor might easily gain the ascendency, and cause the reappearance of long-lost characters.

(Note: Here the translator began the sentence with what Darwin wrote as a clause preceded by another clause in his original English, hence the English is quoted here beginning with a lower-case letter preceded by an ellipse.)

47) Page 525 German, Single vertical line in margin, underlined “gewisse Zahl von Keimchen zur Entwicklung jedes Characters” (“A certain number of gemmules being requisite for the development of each character.”)

Die merkwürdigen, von Mr. Sedgwick hervorgehobenen Fälle von gewissen, regelmässig nur in abwechselnden Generationen auftretenden Krankheiten werden erklärt einmal dadurch, dass eine gewisse Zahl von Keimchen zur Entwickelung jedes Characters nöthig ist, wie es sich darin zeigt, dass mehrere Spermatozoen oder Pollenkörner zur Befruchtung nöthig sind, und dann dadurch, dass die Zeit ihre Vervielfältigung begünstigt. Dies gilt auch mehr oder we-

Page 401 English:
A certain number of gemmules being requisite for the development of each character, as is known to be the case from several spermatozoa or pollen-grains being necessary for fertilisation, and time favouring their multiplication, will together account for the curious cases, insisted on by Mr. Sedgwick, of certain diseases regularly appearing in alternate generations. [Underlining by Mendel.]

48) Page 526 German, single vertical line in margin:

vervielfältigen können. Wir erhalten endlich nach den hier gegebenen Ansichten sicher einen etwas deutlichen Einblick in die wunderbare Thatsache, dass das Kind von dem Typus seiner beiden Eltern abweichen und seinen Grosseltern oder Vorfahren, die durch viele Generationen von ihm getrennt sind, ähnlich sein kann.

Page 402 English:
Finally, on the views here given, we certainly gain some clear insight into the wonderful fact that the child may depart from the type of both its parents, and resemble its grandparents, or ancestors removed by many generations.

49) Page 526 German, single long waving vertical line in margin spanning entire paragraph, with short single straight vertical line, probably highlighting “ist ohne Zweifel ausserst complicirt” (“no doubt is extremely complex”). “Entwickelung hängt” (“development depends”) underlined:

Schluss.

Die Hypothese der Pangenesis, wie sie auf die verschiedenen grossen Classen von Thatsachen, wie sie jetzt erörtert wurden, angewendet wird, ist ohne Zweifel äusserst complicirt. Aber sicher sind es auch die Thatsachen. Die Annahmen indessen, auf denen die Hypothese ruht, kann man nicht als in irgend einem extremen Grad complicirt ansehen, — nämlich, dass alle organischen Einheiten ausser dem Vermögen, was allgemein zugegeben wird, durch Selbsttheilung zu wachsen, noch die Fähigkeit haben, zahlreiche äusserst kleine Atome ihres Inhaltes, d. h. Keimchen abzuwerfen. Diese vervielfältigen und verbinden sich zu Knospen und den Sexualelementen. Ihre Entwickelung hängt von der Vereinigung mit anderen in der Entstehung begriffenen Zellen oder Einheiten ab; und sie sind einer Überlieferung im schlummernden Zustande auf später folgende Generationen fähig.

Page 402 English:
The hypothesis of Pangenesis, as applied to the several great classes of facts just discussed, no doubt is extremely complex; but so assuredly are the facts. The assumptions, however, on which the hypothesis rests cannot be considered as complex in any extreme degree—namely, that all organic units, besides having the power, as is generally admitted, of growing by self-division, throw off free and minute atoms of their contents, that is gemmules. These multiply and aggregate themselves into buds and the sexual elements; their development depends on their union with other nascent cells or units; and they are capable of transmission in a dormant state to successive generations. [Underlining by Mendel.]

50, 51) Page 527 German, two successive single vertical lines in margin:

ihren Vorfahren herrührende schlummernde Keimchen. Diese fast unendlich zahlreichen und kleinen Keimchen müssen in jeder Knospe, in jedem Ei, Spermatozoon und Pollenkorn eingeschlossen sein. Eine solche Annahme wird für unmöglich erklärt werden; aber, wie früher bemerkt wurde, Zahl und Grösse sind nur relative Schwierigkeiten und die von gewissen Thieren und Pflanzen producirten Eier oder Samen sind so zahlreich, dass sie vom Verstand nicht erfasst werden können.

Page 402–403 English:
These almost infinitely numerous and minute gemmules must be included in each bud, ovule, spermatozoon, and pollen-grain. Such an admission will be declared impossible; but, as previously remarked, number and size are only relative difficulties, and the eggs or seeds produced by certain animals or plants are so numerous that they cannot be grasped by the intellect.

52) Page 528 German, single vertical line in margin, "jede Zelle" ("each cell"), "kraft des Umstandes, dass sie von jedem Theil herrührende Keimchen enthält" ("power only in virtue of containing gemmules derived from every part") underlined:

ter und nehme an, dass sie reproductive Keimchen abgeben. Es erzeugt daher ein Thier nicht als ein Ganzes seine Art durch die alleinige Thätigkeit seines Reproductionssystems, sondern jede separate Zelle erzeugt ihre Art. Es haben Naturforscher oft gesagt, dass jede Zelle einer Pflanze die factische oder potenzielle Fähigkeit hat, die ganze Pflanze zu reproduciren. Sie hat dieses Vermögen aber nur kraft des Umstandes, dass sie von jedem Theil herrührende Keimchen enthält. Wird unsere Hypothese provisorisch

Page 403 English:
Thus an animal does not, as a whole, generate its kind through the sole agency of the reproductive system, but each separate cell generates its kind. It has often been said by naturalists that each cell of a plant has the actual or potential capacity of reproducing the whole plant; but it has this power only in virtue of containing gemmules derived from every part. [Underlining by Mendel.]

53) Page 528 German, partial double (first two lines), partial single vertical line, "unzureichende Anzahl von Keimchen innerhalb…aggregirt werden" ("insufficient number of gemmules being aggregated") underlined:

Process, theilweise ausgeführt. Sexuelle Zeugung weicht in mancher wichtigen Hinsicht ab, hauptsächlich, wie es scheinen dürfte, darin, dass hier eine unzureichende Anzahl von Keimchen innerhalb der getrennten Sexualelemente aggregirt werden, und wahrscheinlich noch darin, dass gewisse Primordialzellen vorhanden sind. Die

Page 403 English:
Sexual generation differs in some important respects, chiefly, as it would appear, in an insufficient number of gemmules being aggregated within the separate sexual elements, and probably in the presence of certain primordial cells. [Underlining by Mendel.]

54, 55) Page 528–529 German, double vertical lines, within longer single vertical line in margin on page 529, single vertical line in margin on page 529 continuing sentence from page 528:

Reihenfolge der Entwickelung zunächst kommen. Es sind daher der gewöhnliche Befruchtungsact und die Entwickelung eines jeden Wesens nahe analoge Processe. Streng genommen wächst das Kind nicht zum Mann heran, sondern schliesst Keimchen ein, welche langsam und successiv entwickelt werden und den Mann bilden. Im Kinde erzeugt jeder Theil, ebenso wie im Erwachsenen, denselben Theil für die nächste Generation. Vererbung muss einfach als eine Form von Wachsthum angesehen werden, ebenso wie die Theilung einer niedrig organisirten einzelligen Pflanze. Rückschlag hängt

von der Überlieferung schlummernder Keimchen vom Vorfahren auf seine Nachkommen ab, welche gelegentlich unter gewissen bekannten oder unbekannten Bedingungen entwickelt werden können. Je-

Page 404 English:
[Sentence within double lines:] Thus the ordinary act of impregnation and the development of each being are closely analogous processes. [Remainder within single line:] The child, strictly speaking, does not grow into the man, but includes germs which slowly and successively become developed and form the man. In the child, as well as in the adult, each part generates the same part for the next generation. Inheritance must be looked at as merely a form of growth, like the

self-division of a lowly-organised unicellular plant. Reversion depends on the transmission from the forefather to his descendants of dormant gemmules, which occasionally become developed under certain known or unknown conditions.

56) Page 529 German, double vertical lines in margin:

neuerung jedes lebenden Organismus. So weit mir bekannt ist, ist kein anderer Versuch gemacht worden, so unvollkommen auch der vorliegende ausgesprochenermaassen ist, diese verschiedenen grossen Classen von Thatsachen unter einem Gesichtspunkt zu vereinigen. Wir können die wunderbare Complexität eines organischen

Page 404 English:
No other attempt, as far as I am aware, has been made, imperfect as this confessedly is, to connect under one point of view these several grand classes of facts.

57) Page 529 German, triple vertical lines in margin:

these ist diese Complexität noch bedeutend vergrössert. Jedes lebende Wesen muss als ein Microcosmus betrachtet werden, ein kleines Universum, gebildet aus einer Menge sich selbst fortpflanzender Organismen, welche unbegreiflich klein und so zahlreich sind, wie die Sterne am Himmel.

Page 404 English:
Each living creature must be looked at as a microcosm—a little universe, formed of a host of self-propagating organisms, inconceivably minute and as numerous as the stars in heaven.
